# A Conditional Knockout Mouse Model Reveals That Calponin-3 Is Dispensable for Early B Cell Development

**DOI:** 10.1371/journal.pone.0128385

**Published:** 2015-06-05

**Authors:** Alexandra Flemming, Qi-Quan Huang, Jian-Ping Jin, Hassan Jumaa, Sebastian Herzog

**Affiliations:** 1 Department of Molecular Immunology, Max-Planck-Institut of Immunobiology and Epigenetics, Freiburg, Germany; 2 Biology III, Faculty of Biology, Albert-Ludwigs-University Freiburg, Freiburg, Germany; 3 Department of Medicine, Northwestern University Feinberg School of Medicine, Chicago, Illinois, United States of America; 4 Department of Physiology, Wayne State University School of Medicine, Detroit, Michigan, United States of America; 5 BIOSS Centre for Biological Signalling Studies, Albert-Ludwigs-University Freiburg, Freiburg, Germany; 6 Division of Developmental Immunology, Biocenter, Medical University Innsbruck, Innsbruck, Austria; Ohio State University Comprehensive Cancer Center, UNITED STATES

## Abstract

Calponins form an evolutionary highly conserved family of actin filament-associated proteins expressed in both smooth muscle and non-muscle cells. Whereas calponin-1 and calponin-2 have already been studied to some extent, little is known about the role of calponin-3 under physiological conditions due to the lack of an appropriate animal model. Here, we have used an unbiased screen to identify novel proteins implicated in signal transduction downstream of the precursor B cell receptor (pre-BCR) in B cells. We find that calponin-3 is expressed throughout early B cell development, localizes to the plasma membrane and is phosphorylated in a Syk-dependent manner, suggesting a putative role in pre-BCR signaling. To investigate this *in vivo*, we generated a floxed calponin-3-GFP knock-in mouse model that enables tracking of cells expressing calponin-3 from its endogenous promoter and allows its tissue-specific deletion. Using the knock-in allele as a reporter, we show that calponin-3 expression is initiated in early B cells and increases with their maturation, peaking in the periphery. Surprisingly, conditional deletion of the *Cnn3* revealed no gross defects in B cell development despite this regulated expression pattern and the *in vitro* evidence, raising the question whether other components may compensate for its loss in lymphocytes. Together, our work identifies calponin-3 as a putative novel mediator downstream of the pre-BCR. Beyond B cells, the mouse model we generated will help to increase our understanding of calponin-3 in muscle and non-muscle cells under physiological conditions.

## Introduction

B cell development is initiated in the bone marrow and can be subdivided into distinct stages based on the expression of surface markers and the recombination status of the immunoglobulin (Ig) receptor genes [1]. A major checkpoint is the pre-B cell stage, in which cells that have rearranged the heavy chain gene segments express μ together with the surrogate light-chain components lambda5/VpreB, forming the pre-B cell receptor (pre-BCR) [2]. The pre-BCR promotes survival, proliferation and differentiation of pre-B cells into immature B cells, and thus provides a strong selective advantage. Only cells that receive the appropriate signals are able to maturate further, contributing to the mature B cell pool in the periphery [3].

Signaling from the pre-BCR as well as the BCR results in the activation of the tyrosine kinase Syk, which triggers a cascade of downstream events, ultimately resulting in signaling via the PI3K-PKB axis, in release of intracellular calcium and in consequence the activation of effectors such as protein kinase C (PKC) and transcription factors NF-AT and NF-κB [4–6]. However, although most components in these signaling cascades have been identified in previous efforts, it is tempting to speculate that additional, thus-far unknown proteins may also be functionally relevant.

Calponins form an evolutionary highly conserved family of actin filament-associated proteins expressed in both smooth muscle and non-muscle cells, with three isoforms calponin-1 (basic calponin, h1; encoded by *Cnn1*), calponin-2 (neutral, h2; *Cnn2*) and calponin-3 (acidic, h3, *Cnn3*) in vertebrates [7]. Calponins are characterized by a conserved overall structure, with an N-terminal calponin homology (CH) domain and three calponin repeats (CR) in the C-terminus, and have been shown to bind to a diverse set of molecules including calmodulin, tropomyosin, myosin, desmin, caldesmon, phospholipids as well as to signaling molecules such as extracellular regulated kinase 1/2 (Erk1/2) and protein kinase C (PKC) [8–10]. However, despite structural similarities, calponin isoforms show a distinct pattern of tissue-specific expression, which has raised the question whether they confer individual functions in different cell types.

Calponin-1 is specifically expressed in differentiated smooth muscle cells, where early *in vitro* data indicated that it functions as an inhibitor of the actin-activated myosin ATPase [11]. However, genetic deletion of calponin-1 in mice did not completely abolish muscle function, but rather promoted an early onset of cartilage formation and ossification, increased postnatal bone formation, and accelerated healing of bone fractures, already pointing towards a role of calponins in non-muscle cells [12–14].

Calponin-2 is characterized by a broader tissue distribution, being expressed not only in smooth muscle, but also in several non-muscle tissues [7]. Here, calponin-2 appears to be involved in processes such as cell migration and cell anchorage[15–17]. In mice, for example, calponin-2-deficient macrophages show higher rates of proliferation and faster migration, associated with a significantly increased phagocytotic activity [18]. Interestingly, recent data indicate that gene expression of calponin-2 as well as its stability in these tissues is regulated by mechanical tension in the cytoskeleton, linking external cues to protein function [19,20].

Calponin-3 is the thus far least studied calponin family member. It is expressed in smooth muscle and non-muscle tissue such as the kidney, the lung and the stomach, but has mainly been described in the context of neuronal development and function [21–24]. However, recent data also provide evidence for a function of calponin-3 beyond neurogenesis. In trophoblasts as well as myoblasts, calponin-3 has been identified as a negative regulator of cellular fusion [25,26]. In chondrocytes, it was found to sequester Smad proteins, thereby negatively regulating bone morphogenic protein (BMP)-mediated transcription [27]. Moreover, calponin-3 has been linked to actin stress fiber formation and cell motility during wound healing [28,29]. Nevertheless, despite all these *in vitro* data, little is known about the role of calponin-3 under physiological conditions due to the lack of an appropriate animal model for *in vivo* studies.

Here, we have employed an unbiased screen to identify calponin-3 as a putative component downstream of pre-BCR signaling. Since it has not been described in the context of lymphocytes, we generated a floxed calponin-3-GFP knock-in mouse model and investigated the expression pattern of calponin-3 and its role throughout early B cell development *in vivo*.

## Materials and Methods

### Mass spectroscopy

1.2x10^10^ SLP-65^-/-^ pre-B cells per sample were either left unstimulated or stimulated with 5μl/ml pervanadate/H_2_O_2_ (freshly prepared by mixing 80 μl ddH_2_O with 15 μl 50 mM Na-Orthovanadate solution and 5 μl 30% H_2_O_2_ followed by an incubation on ice for 5 min) for 5 min at 37°C and lysed in 120 ml lysis buffer (50 mM Tris-HCl pH7.4, 137.5 mM NaCl, 1% Glycerol, 1 mM Na-Orthovanadate, 0.5 mM EDTA pH8, 1% digitonin, supplemented with protease inhibitor cocktail, Sigma). Lysates were immunoprecipitated with anti-phosphotyrosine beads (750μl/sample) over night at 4°C. The beads were then transferred to columns, washed with 50 ml lysis buffer, and proteins were eluted by incubating twice with 5 ml 77 mM phenylphosphate (in lysis buffer) for 1 h. Beads were recovered by incubation with 20 ml elution buffer (ImmoPure IgG/IgA Elution buffer, Pierce), and again incubated with supernatant from the previous immunoprecipitation. This procedure was repeated 3 times. Proteins from elutes (30 ml each) were concentrated using Ultrafree protein filters (Merck-Millipore) to a volume of 250 μl. These samples were boiled with reducing sample buffer and separated on a polyacrylamide gel. After Coomassie-staining, bands corresponding to differentially phosphorylated proteins were cut out and identified by mass spectroscopy (carried out by M-scan S.A., USA).

### Cells and cell culture

Primary B cell precursors cells were isolated from the bone marrow of wild type mice, the SLP-65^-/-^ pre-B cell lines *Dec* and *Oct* have already been described [30]. Pre-B cells were cultured in Iscove’s medium (Biochrom) containing 10% fetal calf serum (Vitromex), 2 mM L-glutamine, 100 U/ml penicillin/streptomycin (Biochrom) and 50 μM 2-mercaptoethanol (Gibco) supplemented with excessive IL-7 obtained from the supernatant of stably transfected J558L cells. Schneider S2 cells (a generous gift from Dr. K. Karjalainen, commercially available as CRL-1963 through ATCC) were grown in Schneider’s Drosophila medium (Life Technologies) supplemented with 10% fetal calf serum at 27°C without CO_2_ [4].

### Retroviral constructs and transduction

The retroviral constructs pMOWS-GFP and pMIG have been described [31]. Based on the former, pMOWS-calponin-3-GFP was generated by PCR amplification of *Cnn3* cDNA and in-frame ligation 5’ of the GFP cDNA. Likewise, the calponin-3-GFP deletion constructs were generated by PCR in a way that the proteins lacked aa 2–69 (∆Nterm), aa 143–275 (∆CR) and aa 273–330 (∆Cterm) from the murine calponin-3, respectively. pMIG-HA-calponin-3 was based on pMIG and was generated by cloning a DNA fragment encoding YPYDVPDYA 5’ of the *Cnn3* cDNA.

Retroviral transduction was performed as described [31]. In short, the 293T cell-derived packaging line Phoenix was transfected using GeneJuice (Merck-Millipore) according to the manufacturers instructions. For infection, retroviral supernatants were mixed with polybrene (8 μg/ml final conc.), added to the pre-B cells and centrifuged at 1800 rpm at 37°C for 3 hrs.

### *In vitro* assays

For the immunoprecipitation, 2 x 10^7^ transduced *Dec* pre-B cells per sample were left untreated or were pre-incubated with 1,5 μM Syk inhibitor R406 (Rigel Pharmaceuticals) for 30 min at 37°C. Pre-incubated cells were then left untreated or stimulated with 5 μl/ml pervanadate/H_2_O_2_ for 3 min at 37°C. Cell pellets were lysed in ice-cold lysis buffer (see above, 1% n-Octyl-β-D-glucopyranosid instead of digitonin). HA-tagged proteins were precipitated using anti-HA antibodies (3F10, Roche) and protein-G Sepharose beads (Amersham). Purified samples were washed, mixed with reducing sample buffer, boiled for 10 min and subjected to SDS-PAGE and western blot analysis. For normal visualization of proteins, about 1–2 x 10^6^ cells per sample were directly lysed and used for analysis.

Blotted proteins were detected by anti-calponin 2 (N-18, Santa Cruz), anti-calponin-3 (rabbit polyclonal, generated by Eurogentech), anti-actin (Santa Cruz), anti-GFP (Clontech), anti-phosphotyrosine (4G10, Merck-Millipore) and anti-eIF4alpha antibodies, followed by incubation with HRP-coupled species-specific secondary antibodies (Pierce) and visualization using the ECL system (Amersham).

### Mice

Mb1-cre mice and floxed *Cnn2* mice have been described [18,32]. To generate mice with the floxed calponin-3-GFP mini gene knocked into the endogenous *Cnn3* locus, 129Sv-derived W4 embryonic stem (ES) cells ([33]; a generous gift from Dr. G. Niedermann) were electroporated with 25 μg of the linearized targeting vector (see S1 Supplementary Materials for full sequence and map). ES cells that had integrated the vector were selected with G418 (200 μg/ml) and screened for homologous recombination by Southern blot analysis. In detail, genomic DNA was digested with *BamHI* and hybridized with a 3’ external probe (S1 Supplementary Materials). Positive clones showing the expected band pattern were further verified by PCR as well as by additional digestions (NcoI, HindIII, KpnI) and Southern blot analysis with a 5’ internal and the 3’ external probe (S1 Supplementary Materials), respectively. The correctly targeted ES cell clone was injected into blastocysts and gave rise to chimeric mice which were crossed with CMV-FLPe mice for excision of the neomycin selection cassette. Offspring was screened by PCR analysis (S1 Supplementary Materials) and crossed with CMV-Cre (Balb/c) for deletion of *Cnn3* in all tissues. For calponin-3-GFP expression analyses and for B cell-specific *Cnn3* deletion, *Cnn3* ki f/f mice were backcrossed with C57BL/6 mice for at least 5 generations. In all experiments shown, littermates were compared. Mice were bred at the animal facility of the Max-Planck-Institute of Immunobiology and Epigenetics. Animal studies were carried out in accordance with the German Animal Welfare Act after having been reviewed and approved as project Re-iTO-5 by the institutional animal welfare commission and the local Regional Commission (Regierungspräsidium Freiburg, Referat Veterinärwesen und Lebensmittelüberwachung, Freiburg, Germany).

### Flow cytometry

Single-cell suspensions were prepared from different organs or from transduced cells and stained for FACS analysis using anti-IgM, anti-IgD and anti-B220 (all Southern Biotechnology), anti-CD4, anti-CD8, anti-CD3ε, anti-CD43, anti-CD44, anti-CD19 and anti-CD21 (all BD Pharmingen), anti-CD25 and streptavidin-APC (Biolegend) as well as streptavidin-Cy5/PerCP (Dianova). Data were accquired on a FACSCalibur, LSRII (Becton Dickinson) or CyAn ADP Analyzer (Beckman Coulter) and analyzed with FlowJo software (Tree Star). For measurement of calcium release upon stimulation, about 1–2 x 10^6^ cells derived from the bone marrow or the spleen were incubated with 5 μg/ml of Indo-1 AM (Molecular Probes) and 0.5 μg/ml of pluronic F-127 (Molecular Probes) in Iscove’s medium supplemented with 1% FCS at 37°C. After 45 minutes, cell pellets were resuspended in normal growth medium, and the Ca^2+^ flux was induced by addition of 10 μg/ml of goat anti-mouse kappa (Southern Biotechnology) or by addition of 5 μl/ml freshly prepared pervanadate mix. Events were recorded for 6–8 min after stimulation.

### RT-PCR analysis

Total RNA was isolated from the *Oct* pre-B cell line as well as from precursor B cells derived from the bone marrow of a *Cnn3* f/f ki mouse or a littermate control after 5 days of culture in IL-7 supplemented medium. 1 μg of RNA was reverse-transcribed into cDNA using the First Strand cDNA Synthesis Kit (Fermentas) according to the manufacturers’ instructions. For the endpoint PCR, cDNA fragments were amplified using as CCGCTGGGGCTAAGAG a forward primer and ATGATGCCGTCCTTCAGC (PCR1) or TGAACTTGTGGCCGTTTACGTC (PCR2) as reverse primers, respectively.

### Statistical analysis

Statistical analyses were carried out using the GraphPad prism software 5 (GraphPad Software, La Jolla, CA). Groups of two were compared by unpaired or paired t-tests, respectively (see figure legends for details). Statistical significance was defined as p≤0.05. Values are expressed as mean ±SD.

## Results

### Calponin-3 becomes phosphorylated upon stimulation of B cell precursors

**Fig 1:**
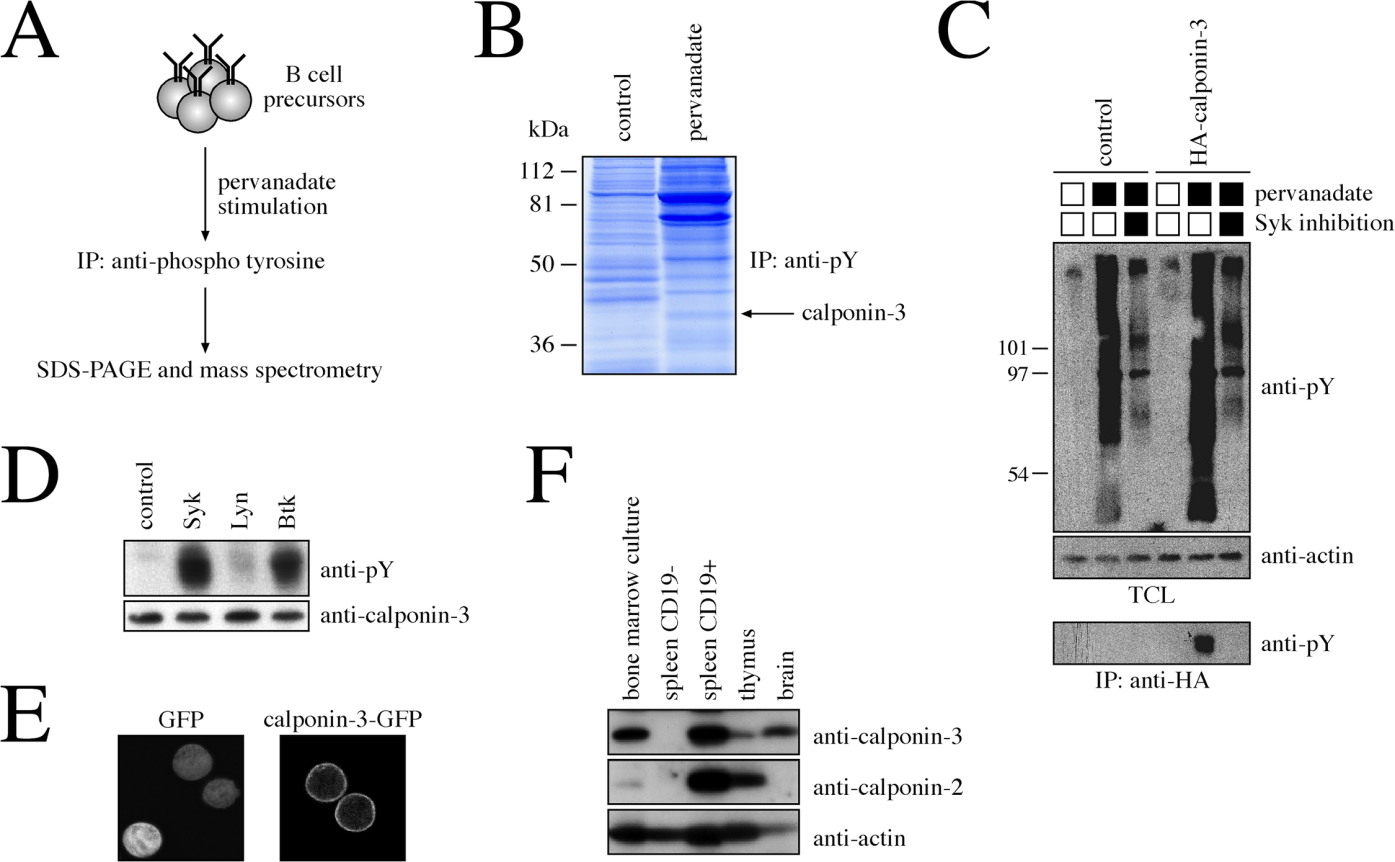
Calponin-3 is phosphorylated upon stimulation of B cell progenitors. A. Schematic illustration of the conducted screen designed to identify signaling components downstream of the pre-B cell receptor. B. Coomassie Blue staining of an SDS-PAGE showing constitutive (control) and pervanadate-induced tyrosine-phosphorylation of proteins in B cell progenitors. The position of the band corresponding to calponin-3 is marked by an arrow. C. Western blot indicating Syk-dependent phosphorylation of calponin-3 upon pervanadate stimulation in B cell progenitors. Pre-B cells transduced with an empty control vector or a with a vector encoding an HA-tagged calponin-3 were stimulated with pervanadate in the presence or absence of a Syk inhibitor for 3 min. Untreated cells served as a control. Cellular lysates either directly subjected to SDS-PAGE and western blotting or immunoprecipitated with an anti-HA antibody. Actin was used as a loading control. D. Western blot analysis for tyrosine phosphorylation of calponin-3 in the S2 Schneider cell system. S2 cells were transfected with expression constructs encoding calponin-3 and Syk, Lyn or Btk, respectively. Cellular lysates were subjected to SDS-PAGE and western blotting. E. Confocal image of pre-B cells expressing GFP or a calponin-3-GFP fusion protein, respectively. F. Western blot for analysis of calponin 2 and 3 expression in bone marrow cells cultured with IL-7 for 5d, in sorted CD19- and CD19+ splenic B cells, in total thymocytes and in the brain. Western blotting against actin was used as a loading control.

In order to identify novel signaling components downstream of the pre-BCR, we established an unbiased screen for proteins that become tyrosine-phosphorylated upon stimulation of B cell precursors (Fig 1A). In detail, SLP-65^-/-^ pre-B cells, which express high levels of the pre-BCR and are arrested at the pre-B cell stage due to lack of the adapter protein SLP-65 [34], were left untreated as a control or treated with the protein tyrosine phosphatase inhibitor pervanadate, mimicking strong receptor activation. Following cell lysis, tyrosine-phosphorylated proteins were immunoprecipitated, subjected to SDS-PAGE and visualized by Coomassie Blue staining. Bands corresponding to differentially phosphorylated proteins were cut out and analyzed by mass spectrometry (Fig 1B). One of the proteins that showed strong inducible phosphorylation upon pervanadate stimulation was calponin-3.

As calponin-3 has been implicated in cytoskeletal organization and signaling, but not in the context of lymphocytes, we decided to investigate its role in B cells in more detail. To verify our initial finding of inducible tyrosine phosphorylation, pre-B cells expressing an HA-tagged calponin-3 or an empty vector as a control were first stimulated with pervanadate for 3 min, either in the presence or in the absence of the Syk kinase inhibitor R406 (Fig 1C). In accordance with the initial screen, immunoprecipitation and western blot analysis showed a strong phosphorylation of calponin-3 in stimulated pre-B cells. However, concomitant treatment of cells with R406 almost completely abolished overall as well as specific calponin-3 tyrosine phosphorylation. In the S2 Schneider cell system [4], which allows to study the biochemical interplay of foreign proteins in the genetically distant environment of Drosophila, co-transfection of calponin-3 with Syk and its downstream kinase Btk, but not with the Src kinase Lyn, resulted in a strong phosphorylation of calponin-3 (Fig 1D). Furthermore, confocal microscopy of pre-B cells indicated the localization of a calponin-3-GFP fusion protein to the plasma membrane, and thus to the intracellular compartment where pre-BCR signaling is initiated (Fig 1E). This membrane association required the calponin repeats as well as the N-terminal region comprising parts of the calponin homology domain, whereas the C-terminal acidic tail was dispensable (S1 Fig). Lastly, western blot analysis revealed strong expression of calponin-3 in primary B cell precursors as well as in mature B cells, albeit to slightly lower levels compared to the brain (Fig 1F). In contrast, calponin-3 was undetectable in non-B cells of the spleen, whereas thymic cells seemed to express low amounts. Family member calponin-2 was abundant in the thymus and in splenic B cells, but only weakly expressed in B cell precursors (Fig 1F), whereas calponin-1 was not detectable in any of the analyzed cell types (data not shown). Taken together, this indicates that calponin-3 is specifically expressed in early B lymphocytes, localizes to the plasma membrane and becomes tyrosine phosphorylated in a Syk-dependent manner upon stimulation of B cell precursors.

### Targeting of the *Cnn3* locus

**Fig 2:**
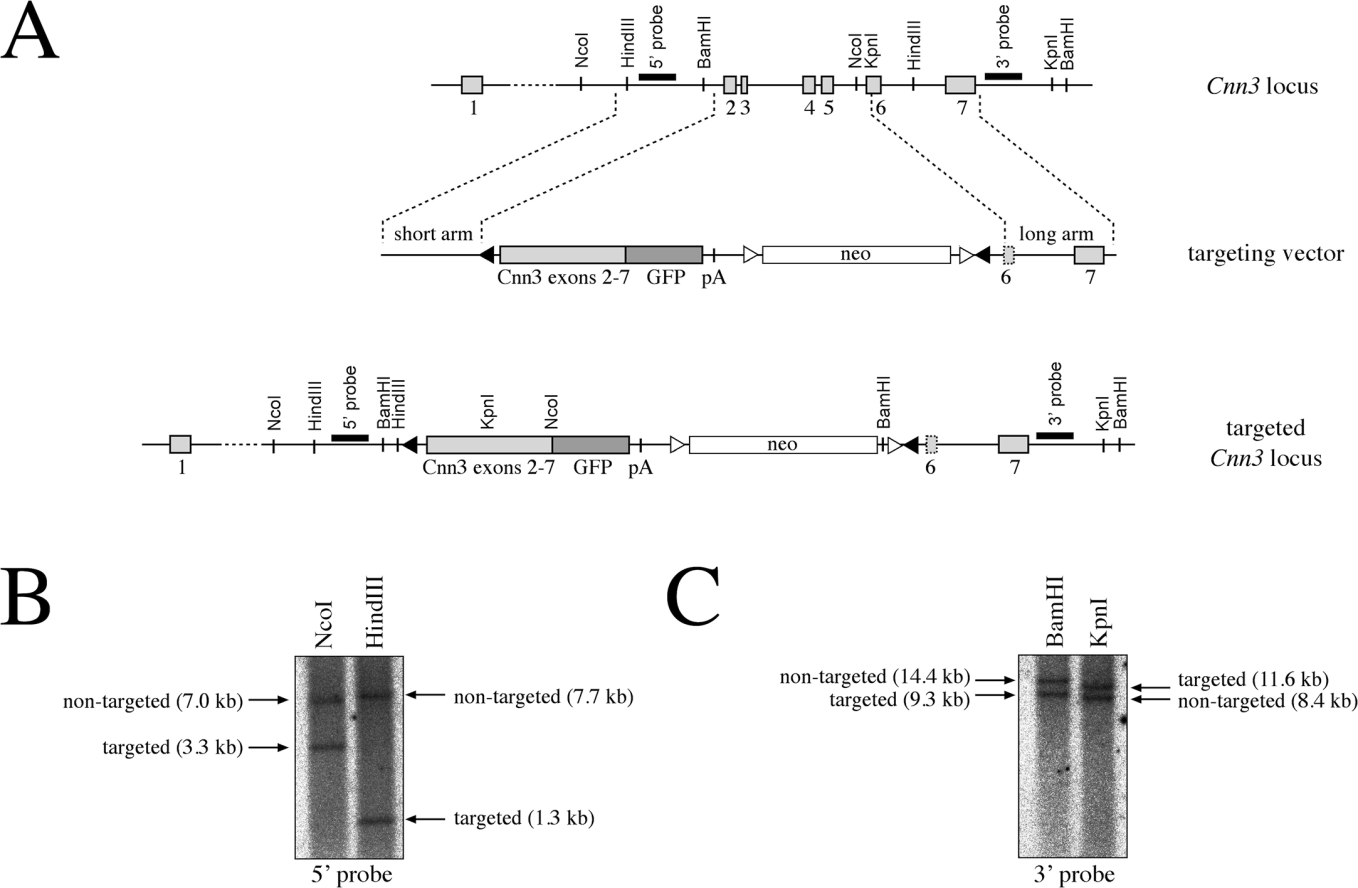
Targeting of ES cells to generate a floxed calponin-3-GFP knock-in. A. Schematic illustration of the *Cnn3* locus, the targeting construct and the *Cnn3* locus after targeting. The targeting strategy aimed at replacing exons 2 to 5 with a floxed mini gene corresponding to exons 2 to 7 fused to a GFP-cDNA. Exons and the mini gene are represented by grey boxes, the neomycin-resistance gene is illustrated by a white box. LoxP-sites are depicted as black triangles, FRT-sites as white triangles. Restriction sites for the enzymes used for southern blot analysis are indicated. Please note that the illustration is not in scale. B. Southern blot analysis of the targeted clone used for blastocyst injection. Genomic DNA was digested by NcoI, HindIII, BamHI or KpnI, respectively, separated by agarose gel electrophoresis and blotted. The blot was hybridized with the internal 5’- and the external 3’-probe as indicated in A. Arrows indicate the positions of fragments corresponding to the non-targeted as well as the targeted allele.

Based on our initial screen and the *in vitro* analyses in pre-B cells, we were wondering whether calponin-3 plays a role in early B cell development. To investigate this *in vivo*, a targeting vector was constructed where *Cnn3* exons 2 to 5 were replaced by a floxed mini gene comprising *Cnn3* exons 2 to 7, deleted for the stop codon and fused to a GFP cDNA (Fig 2A). Following splicing from the endogenous exon 1 to the mini gene, this was expected to result in expression of a floxed full-length calponin-3-GFP fusion protein under control of the endogenous promoter and its regulatory elements. This strategy allowed for a dual application: First, conditional Cre-mediated deletion of the mini gene generates a null allele, enabling the tissue-specific analysis of cellular function in the absence of calponin-3. Second, targeted mice serve as a fluorescent reporter to track cells and tissues for calponin-3 expression *in vivo*. Of note, a corresponding calponin-3-GFP fusion protein was tested beforehand in pre-B cells and displayed the same pervanadate-induced phosphorylation as the HA-tagged calponin-3, suggesting that the C-terminal GFP tag does not compromise protein function (data not shown).

A 129S6/SVEvTac ES cell line was transfected with the targeting vector, and from about 580 clones growing under G418 selection, 6 clones were positive for the correct integration by Southern blot analysis with a 3’ external probe. However, detailed analysis by additional Southern blot hybridizations, PCR and sequencing revealed that only one of these clones was correctly targeted at the 5’ end and contained the 5’ loxP site (Fig 2B and data not shown). When injected into C57BL/6 blastocysts, this clone produced chimeric mice that were crossed with FLPe-transgenic mice to remove the gene encoding neomycin resistance. Germ line transmission of the knock-in and deletion of the *neo* gene were confirmed by PCR (data not shown). Heterozygous intercrosses of calponin-3-GFP mice (+/f) generated the expected frequencies of transmission of the floxed allele, and homozygous mice (*Cnn3* ki f/f) appeared healthy.

### Expression of calponin-3-GFP from the endogenous *Cnn3* locus

**Fig 3:**
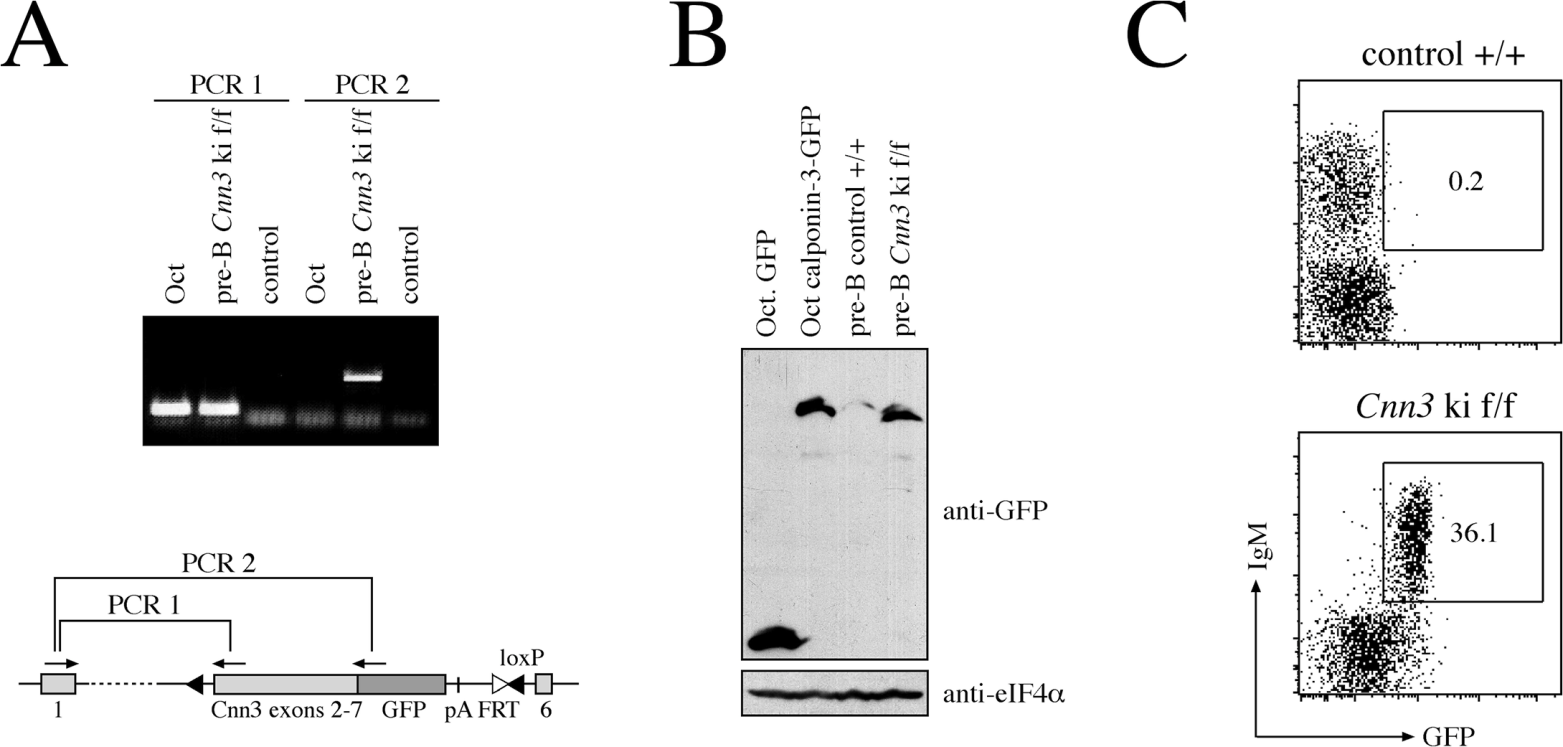
Calponin-3-GFP is expressed from the endogenous *Cnn3* locus. A. RT-PCR analysis of the *Cnn3* mRNA splicing in targeted cells. Total RNA from the pre-B cell line Oct as well as from pre-B cells derived from a *Cnn3* ki f/f mouse was transcribed into cDNA and analyzed by PCR. Primer pairs for PCRs 1 and 2 are depicted in the schematic illustration of the targeted allele. A sample lacking cDNA served as a control. B. Western blot analysis for expression of the calponin-3-GFP fusion protein from the targeted *Cnn3* locus. Pre-B cells derived from a *Cnn3* ki f/f mouse or a non-targeted littermate were lysed and subjected to SDS-PAGE and blotting. Oct pre-B cells expressing GFP and calponin-3-GFP were used as a control. Equal loading was demonstrated by anti-eIF4α. C. Flow cytometric analysis of cells from a *Cnn3* ki f/f mouse or a non-targeted +/+ littermate. Cells were isolated from the bone marrow of the respective mice, stained with an anti-IgM antibody and analyzed for expression of IgM and GFP by flow cytometry. Numbers indicate the percentage of cells in the respective gate.

To test whether the abovementioned targeting strategy indeed promotes the expression of calponin-3-GFP from the endogenous promoter, bone marrow-derived pre-B cells from homozygous knock-in mice (*Cnn3* ki f/f) were analyzed by RT-PCR. Using non-targeted pre-B cells as a control, we were able to confirm that exon 1 is indeed correctly spliced to the mini gene, giving rise to a transcript encoding full-length calponin-3-GFP (Fig 3A). In correspondence with this, western blot analysis of the same cells showed a band at the same height as pre-B cells stably expressing a full-length calponin-3-GFP construct (Fig 3B). Moreover, GFP fluorescence from the fusion construct was readily detected by flow cytometry in B cells isolated from the bone marrow of *Cnn3* ki f/f mice (Fig 3C), supporting the initial protein expression data (Fig 1F). Beyond B cells, calponin-3-GFP expression was also clearly visible in *Cnn3* ki f/f embryos using fluorescence imaging, showing a broad expression pattern throughout early development (S2 Fig). Together, these data suggest that the knock-in mouse model is suitable for monitoring calponin-3 expression in different tissues and in response to different stimuli.

### Calponin-3 is expressed throughout B cell development, but is restricted to a subset of thymic T cells

**Fig 4:**
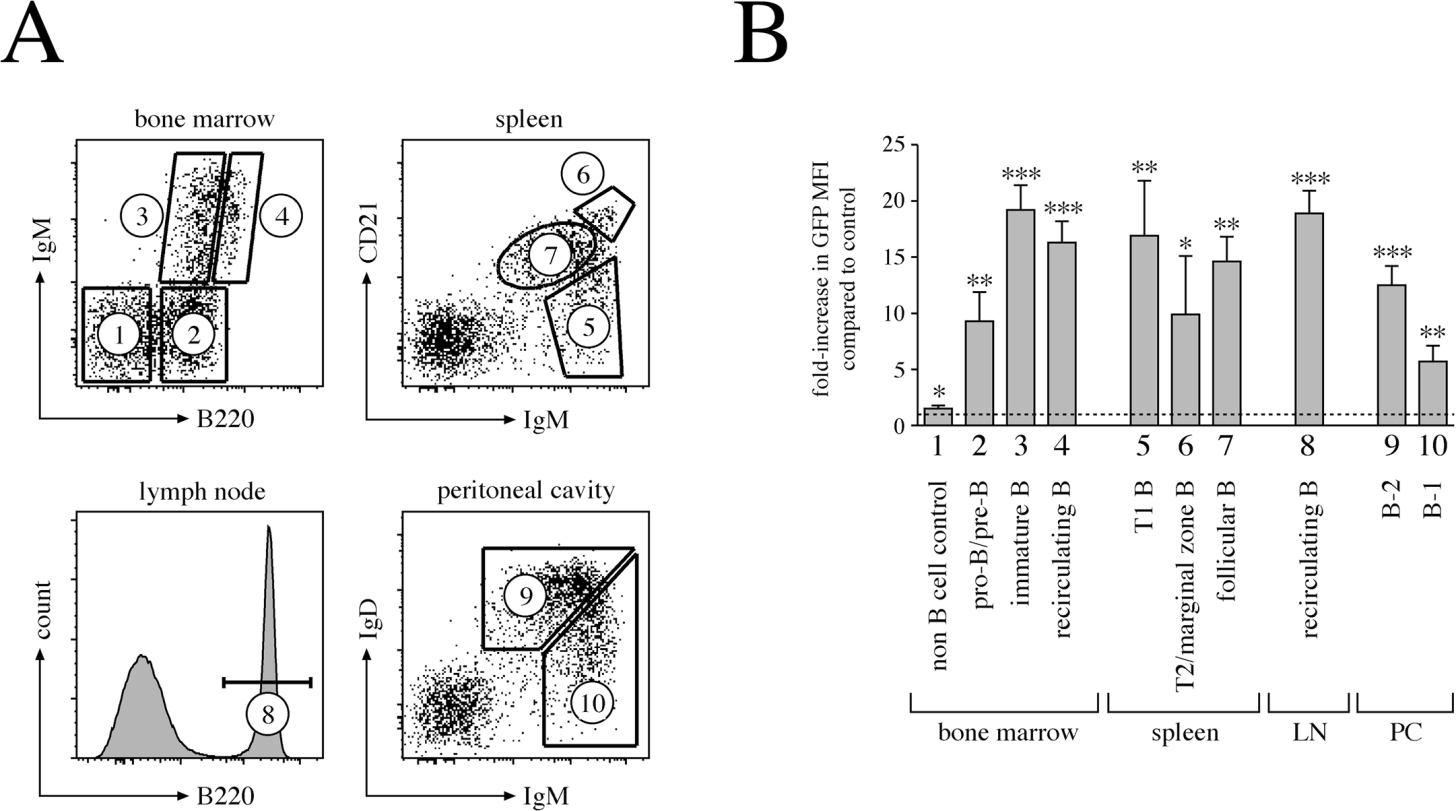
High expression of calponin-3 throughout B cell development. A. Staining pattern and gating strategies for cells isolated from the bone marrow, the spleen, lymph nodes and the peritoneal cavity. Numbers indicate the respective populations as analyzed in B. Expression of calponin-3-GFP in different B cell subsets derived from a *Cnn3* ki f/f mouse or a +/+ littermate. Cells were isolated, stained and analyzed as indicated in A. Bar graphs indicate the ratio of the GFP MFI of ki f/f cells versus that of +/+ cells. Data represent 4 independent experiments. For statistical analysis, normalized GFP MFI values of control and ki f/f littermates were compared by a paired t-test (p≤0.05 = *, p≤0.01 = **, p≤0.001 = ***). LN, lymph node; PC, peritoneal cavity.

In order to determine the level of calponin-3 protein expression in the different developmental subpopulations of B cells, we analyzed cells isolated from the bone marrow, the spleen, lymph nodes and the peritoneal cavity of *Cnn3* ki f/f and control mice, respectively, by flow cytometry (Fig 4A). Using the change in GFP mean fluorescence intensity (MFI) as a read out, our reporter mouse revealed a clear expression of calponin-3-GFP already in pro-/pre-B cells in the bone marrow, with even higher levels in immature and mature B cells (Fig 4B). In contrast, non-B cells (defined as negative for B220) showed no calponin-3-GFP fluorescence, recapitulating the western blot analysis (Fig 1F). In the spleen, calponin-3 expression was equally high in splenic transitional 1 and follicular B cells, with only the pool of marginal zone and transitional 2 B cells appearing slightly lower in GFP fluorescence (not statistically significant). In the periphery, B cells isolated from lymph nodes retained high calponin-3 levels, whereas peritoneal cavity B-2 B cells and even more B-1 B cells showed reduced expression. Compared to the B cell compartment, calponin-3-GFP expression in T cells was in general weaker and restricted to the early developmental subsets (S3 Fig).

### Calponin-3 is dispensable for early B cell development

**Fig 5:**
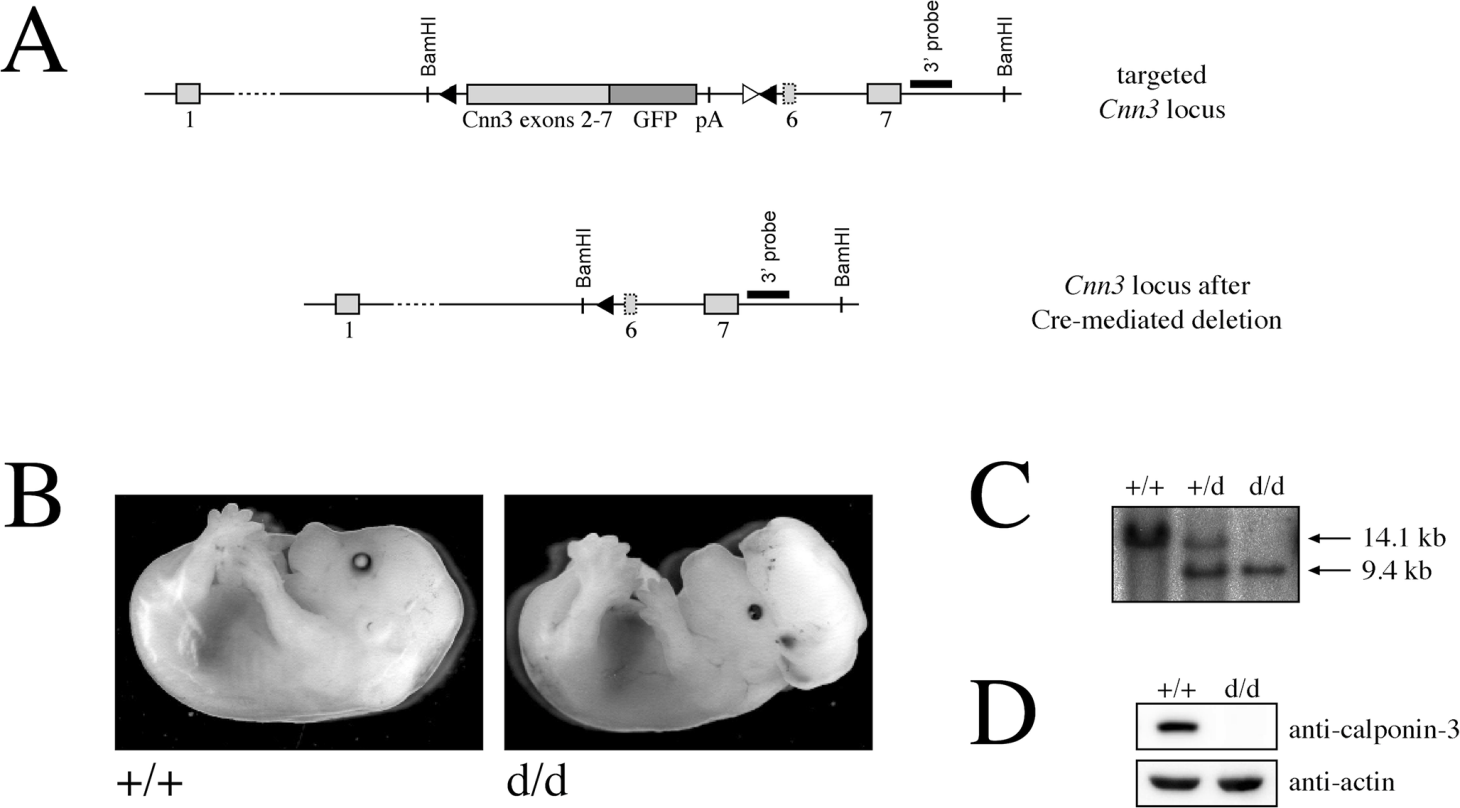
Deletion of calponin-3 results in exencephaly. A. Targeted *Cnn3* locus before and after Cre-mediated deletion, illustrated as in Fig 2A. B. Reflected light images of *Cnn3* ko d/d embryos their +/+ littermates as a control. C. Southern blot analysis of a control mouse (+/+), a heterozygous *Cnn3* (+/d) and a homozygous *Cnn3* knockout mouse (d/d). BamHI-digested genomic DNA from the respective mice was separated by gel electrophoresis, blotted and hybridized with the 3’ external probe. Arrows indicate the positions of fragments corresponding to the wild-type (+) as well as the deleted (d) allele. D. Fetal brain tissue of a homozygous *Cnn3* knockout and a control littermate was lysed, subjected to SDS-PAGE and western blotting and probed for calponin-3 and actin expression as a control.

**Fig 6:**
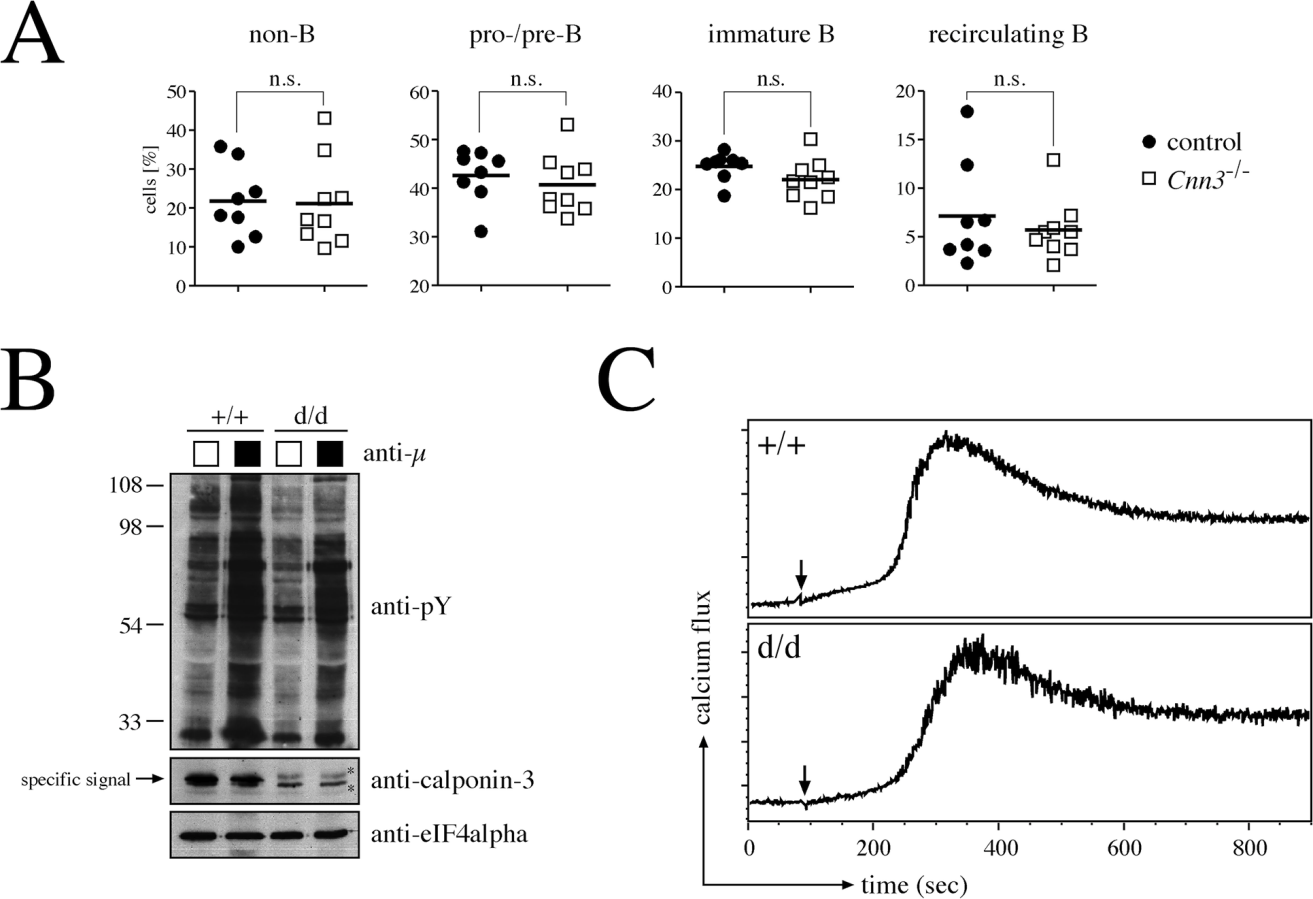
Conditional deletion of calponin-3 does not affect early B cell development and function. A. Percentages of different developmental stages and cell types derived from the bone marrow of control and B cell-specific *Cnn3* knockout mice. Staining and gating of cells were performed according to Fig 4A. Control littermates (+/+ or +/f, positive for mb1-Cre) are depicted as black dots, knockout animals (f/d or f/f, positive for mb1-Cre) as white squares. Black bars mark the averaged percentage of cells for each subgroup. Percentages of cells in control and knockout animals were compared in an unpaired t-test (p>0.05 = not significant, n.s.). B. Western blot indicating induced signaling of control and calponin-3-deficient cells upon pre-BCR crosslinking. BM-derived B cells were starved for 30 min and then stimulated with an anti-μ antibody for 3 min. Cellular lysates were subjected to SDS-PAGE and western blotting. Anti-actin was used as a loading control, anti-calponin-3 confirmed the genotype of the used cultures. The band corresponding to calponin-3 is marked by an arrow, whereas non-specific signals from the polyclonal anti-calponin-3 antibody are labeled with asterisks. C. Induced calcium flux in B cell precursors derived from the bone marrow of control and knockout mice. Bone marrow cells cultured in the presence of IL-7 for 5 d were loaded with Indo-1, stimulated with pervanadate (marked by arrow) and analyzed by flow cytometry.

Together, our *in vitro* results and the *in vivo* expression pattern suggested a putative role of calponin-3 in early B cell development. To test this, we crossed our *Cnn3* ki f/f mice with a CMV-Cre transgenic strain to excise the floxed mini gene, thereby generating a null allele (Fig 5A). However, homozygeous calponin-3 knockout mice (d/d) displayed an extensive growth of neuronal tissue during early embryonic development, were born with a severe exencephaly and died immediately postnatal (Fig 5B to 5D, manuscript in preparation). To enable investigation of calponin-3 in the B cells, we hence restricted deletion of the *Cnn3* gene to the B cell compartment by crossing our mice with the mb1-cre mouse strain. Percentages of non-B cells, pro-/pre-B cells, immature and recirculating mature B cells in the bone marrow of these mice were comparable to that of controls (Fig 6A). Moreover, when stimulated with an antibody that crosslinks the pre-BCR, overall induced tyrosine phosphorylation as measured by western blotting was identical for both genotypes (Fig 6B). Furthermore, the kinetic of induced calcium flux in *Cnn3* ko d/d versus littermate control cells was comparable (Fig 6C). Reflecting this normal functional capacity and distribution of cells in the bone marrow, we were furthermore unable to find any statistically significant differences in the percentages and the signaling capacity of splenic B cells as measured by induced calcium flux (S4 Fig). This suggests that calponin-3, albeit highly expressed, is either not functionally involved in early B cell development or that its loss can be compensated, e.g. by calponin 2. To rule this out, we crossed our *Cnn3* ki f/f mb1-cre mice with a floxed *Cnn2* strain previously described [18]. However, the B cell-specific deletion of calponin 2 and 3 did not affect the formation of precursor and mature B cell populations (S5 Fig), indicating that calponins are dispensable for B cell development.

## Discussion

In this study, we have used an unbiased screen to identify calponin-3 as a putative signaling component downstream of the pre-BCR. In particular, we have shown that calponin-3 is associated with the plasma membrane, and thus is located in the direct vicinity of the pre-BCR signal transduction machinery in B cell precursors. Treatment of pre-B cells with pervanadate, which mimics strong receptor activation by shifting the kinase-phosphatase equilibrium towards the former, resulted in strong tyrosine phosphorylation of calponin-3 depending on the activity of the Syk kinase. Co-expression of calponin-3 with various kinases in S2 Schneider cells promoted a phosphorylation of calponin-3 by Syk, but also by the Tec family kinase Btk. It has been shown that Btk requires phosphorylation by Syk to become fully activated [35], which may explain why calponin-3 phosphorylation was completely abolished upon Syk inhibition. Whether calponin-3 is directly phosphorylated by just one or by both kinases in our experimental system is unclear, but a concerted action of Syk and Btk in a complex of signaling proteins as described for the activation of phospholipase C-γ2 appears likely [36].

Until now, expression of calponin-3 has been described in various tissues and cell types [21,22,25–28,37,38]. In this light, it is not surprising that we find calponin-3 also in lymphocytes, reflecting that calponin-3 and most likely also calponin-2 play roles in a diverse set on non-muscle cells. Indeed, calponin-2 has been described in the context of myeloid cells [18] and is, as we have shown here, also expressed in splenic B cells. Using our knock-in mouse as a reporter, we have been able to precisely locate the developmental B cell and T cell stages in which calponin-3 is expressed *in vivo*. Whereas calponin-3-expression appears to be restricted to thymic T cells, B cells display an intermediate GFP fluorescence already in the bone marrow pro-B/pre-B compartment, but significantly higher levels in immature and mature B cells. This implies that calponin-3 expression is turned on early and peaks in later stages, suggesting a possible role in both B cell development and immune function. However, conditional deletion of calponin-3 in pro-B using mb1-cre mice did not severely affect B cell development, nor did we observe any defects in overall signaling and calcium flux.

Based on its expression pattern and its structural properties, with its ability to bind cytoskeletal elements on the one hand, and molecules like Erk1/2, Smad and PKCs on the other hand, we hypothesized that calponin-3 might be a factor downstream of pre-BCR signaling. Thus, we can only speculate about the lack of an obvious phenotype in the B cell-specific *Cnn3* ^-/-^ mice. First, it may be that cells lacking calponin-3 are functionally impaired with respect to signaling, but that this does not manifest in an alteration of the developmental stages or calcium mobilization. However, this is unlikely, as aberrant signaling from the pre-BCR has profound effects in the bone marrow and in the periphery [39,40]. Defective activation of Erk1/2, for example, is associated with reduced cell expansion and a block at the pro-B to pre-B cell transition [41]. Likewise, loss of PKC-mediated signaling strongly affects normal B cell development [42,43]. Second, it is possible that other family members, or other proteins with similar structural properties, may be able to compensate for the loss of calponin-3. Given that we did not find any calponin-1 expression in B cells, a likely candidate was calponin-2. However, B cell development in *Cnn2*/*Cnn3*-double-deficient mice was also not significantly altered, arguing against a compensatory effect at least within the calponin family. A third possibility is that calponin 3 plays an inhibitory role, rather than being an activator that links receptor stimulation to downstream signaling events. As such, sequestration of molecules such as Erk1/2 or PKCs to calponin 3, and phosphorylated calponin 3 in particular, may counteract their normal function and thus may establish a negative feedback loop that limits cellular signaling. So far, we have not observed any signs of such an inhibitory function of calponin 3, and mice monitored for up to one year did not develop any signs of autoimmunity as a consequence of a putative cellular hyperactivation. However, it is possible that loss of an inhibitory calponin 3 may not lead to a severe phenotype under steady-state conditions, warranting a detailed analysis in a challenging context, e.g. in an autoimmune-prone mouse strain. Moreover, it might be interesting to define the interactome of calponin 3 in lymphocytes, both in its non-phosphorylated and in its phosphorylated state, to undercover such a possible decoy function. Fourth, it is possible that the phosphorylation of calponin-3 induced by pervanadate stimulation, although widely used in the signaling field to mimic strong receptor engagement, does not reflect a physiological condition. Despite intense efforts, we have not been able to detect calponin-3 phosphorylation upon stimulation with an anti-μ antibody, which may be considered a “cleaner” way to trigger pre-BCR signaling. On the other hand, the S2 Schneider cell data clearly demonstrate that kinases involved in pre-BCR signaling can in principle bind and phosphorylate calponin-3. Thus, it may very well be that phosphorylation under physiological conditions takes place and is functionally relevant, but is below the detection limit. Indeed, although the role of phosphorylation for the regulation of calponins has been established in numerous *in vitro* experiments, the detection of phospho-calponins under physiological *in vivo* conditions has been difficult [44].

In contrast to the conditional deletion of calponin-3 in B cells, the whole body knockout animals showed a strong neuronal phenotype, which is in correspondence with the high expression of calponin-3 in neuronal tissue [22,23]. Such a phenotype has already been proposed based on the high expression of calponin-3 in the early stages of hippocampal development compared to its low expression in adult hippocampi, suggesting a role in neural cell proliferation and migration [38]. Whether loss of calponin-3 also affects neural plasticity, as has been postulated based on *in vitro* data [24], needs to be investigated in the future.

Besides the neuronal phenotype, embryos appeared to be morphologically normal, which is surprising given the broad expression pattern of calponin-3-GFP observed in the knock-in animals, e.g. in the skin. However, we cannot rule out that other calponin family members may be able to compensate the loss of calponin-3 in these tissues. Whether deletion of calponin-3 affects trophoblast fusion, myoblast fusion or wound healing *in vivo*, as has been suggested [25,26,28], remains to be determined.

In summary, here we have identified calponin-3 as a putative element downstream of pre-BCR signaling. Deletion of calponin-3 in B cells, however, did not lead to a profound phenotype, arguing against an important role in B cell development. Nevertheless, the mouse model we generated in the course of this project will be of benefit for assessing the role of calponin-3 under physiological conditions. It not only simplifies the identification and tracking of calponin-3-expressing cells *in vivo*, but also allows its analysis in a loss-of-function approach. Crossing our mice with a Cre-transgenic strain of choice will reveal the tissue-specific function of calponin-3, and thus will help to shed light on the role of calponin-3 in muscle and non-muscle cells.

## Supporting Information

S1 Fig The calponin homology domain and the calponin repeats, but not the C-terminal tail, are indispensable for membrane localization of calponin-3-GFP in B cell progenitors. Confocal image of pre-B cells expressing GFP, a full-length calponin-3-GFP fusion protein as well as mutants lacking the N-terminal region comprising parts of the calponin homology domain (∆Nterm), the calponin repeats (∆CR) or the acidic C-terminus (∆Cterm), respectively. Schematic illustrations of the used GFP-fusion constructs are depicted on the left.
(PDF)

S2 Fig Expression of calponin-3-GFP from the endogenous *Cnn3* locus throughout embryogenesis. Reflected light images (left) and GFP fluorescence (right panels) in *Cnn3* ki f/f and +/+ embryos.
(PDF)

S3 Fig Low calponin-3 expression in T cells. A. Staining pattern and gating strategies for cells isolated from the thymus, lymph nodes, the spleen and the peritoneal cavity. For the identification of early T cell progenitors, thymic cells were stained for CD3e, B220, CD4 and CD8, and lineage-negative cells were further subdivided according to their expression of CD44 and CD25, respectively. Numbers indicate the respective populations as analyzed in B. T cell expression of calponin-3-GFP in different tissues and different developmental stages derived from a *Cnn3* ki f/f mouse or a +/+ littermate. In analogy to Fig 4, bar graphs depict the ratio of the GFP comparing ki f/f cells versus +/+ cells. Data represent 4 independent experiments. For statistical analysis, normalized GFP MFI values of control and ki f/f littermates were compared by a paired t-test (p>0.05 = not significant (n.s.), p≤0.05 = *, p≤0.01 = **, p≤0.001 = ***). LN, lymph node; PC, peritoneal cavity; SP, single-positive; DP, double-positive.
(PDF)

S4 Fig Deletion of calponin-3 does not affect splenic B cell populations and calcium signaling. A. Percentages of different developmental stages and cell types derived from the spleen (according to Fig 4A) of control and B cell-specific Cnn3 knockout mice. Controls (+/+ or +/f, positive for mb1-Cre) are depicted as black dots, knockout animals (f/d or f/f by tail PCR, positive for mb1-Cre) as white squares. Individual percentages are calculated on basis of IgM-positive cells. Black bars mark the averaged percentage of cells for each subgroup. Percentages of cells in control and knockout animals were compared in an unpaired t-test (p>0.05 = not significant, n.s.). B. Induced calcium flux in splenic B cells isolated from control and knockout mice. Cells were counterstained with anti-CD43 to exclude T cells, loaded with Indo-1, stimulated with anti-kappa (marked by arrow) and analyzed by flow cytometry.
(PDF)

S5 Fig Simultaneous deletion of calponin 2 and calponin-3 does not impair early B cell development. Comparison of bone marrow (upper row) and splenic (lower row) B cell populations in a calponin 2/calponin-3-double deficient mouse (f/f,f/f,mb1-cre^+^) compared to a calponin 2-deficient (f/f,+/f,mb1-cre^+^) and a wild type littermate (f/f,+/+). Numbers indicate the percentage of cells in the respective region.
(PDF)

S1 Supplementary Materials Supplementary information about the targeting vector, southern blot probes and the genotyping strategy. (PDF)
