# Supplementary material for: A Conditional Knockout Mouse Model Reveals That Calponin-3 Is Dispensable for Early B Cell Development
Source: PLoS One. 2015 Jun 5;10(6):e0128385. doi: 10.1371/journal.pone.0128385 (PMC4457629; doi:10.1371/journal.pone.0128385)
Supplement: S1 Fig — Confocal image of pre-B cells expressing GFP, a full-length calponin-3-GFP fusion protein as well as mutants lacking the N-terminal region comprising parts of the calponin homology domain (∆Nterm), the calponin repeats (∆CR) or the acidic C-terminus (∆Cterm), respectively. Schematic illustrations of the used GFP-fusion constructs are depicted on the left. (PDF) [file pone.0128385.s001.pdf]

Figure S1

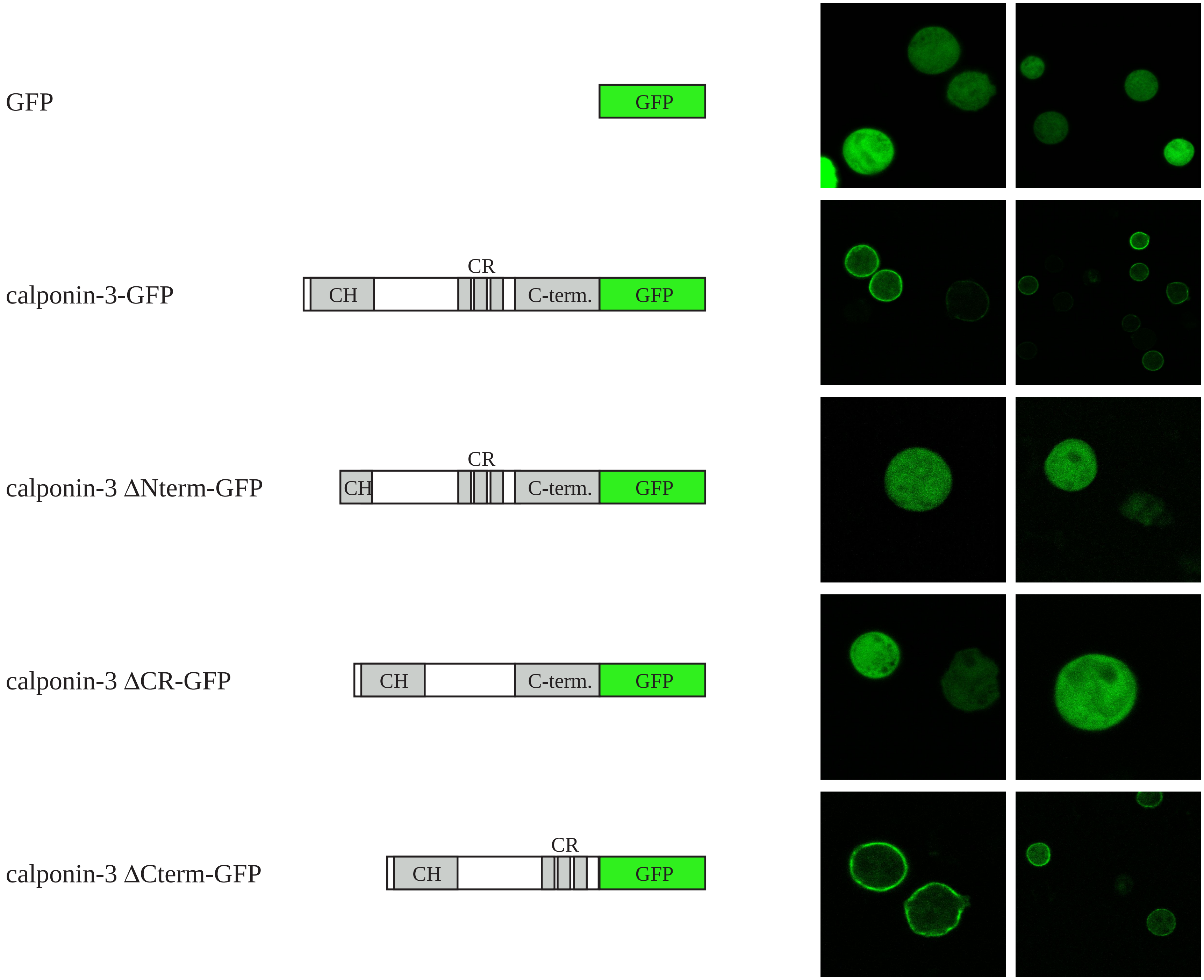
GFP
GFP
calponin-3-GFP
CR
CH
C-term.
GFP
calponin-3 ΔNterm-GFP
CR
CH
C-term.
GFP
calponin-3 ΔCR-GFP
CH
C-term.
GFP
calponin-3 ΔCterm-GFP
CR
CH
GFP
