# Supplementary figures and images for: A Conditional Knockout Mouse Model Reveals That Calponin-3 Is Dispensable for Early B Cell Development
Source: PLoS One. 2015 Jun 5;10(6):e0128385. doi: 10.1371/journal.pone.0128385 (PMC4457629; doi:10.1371/journal.pone.0128385)

Figure S2

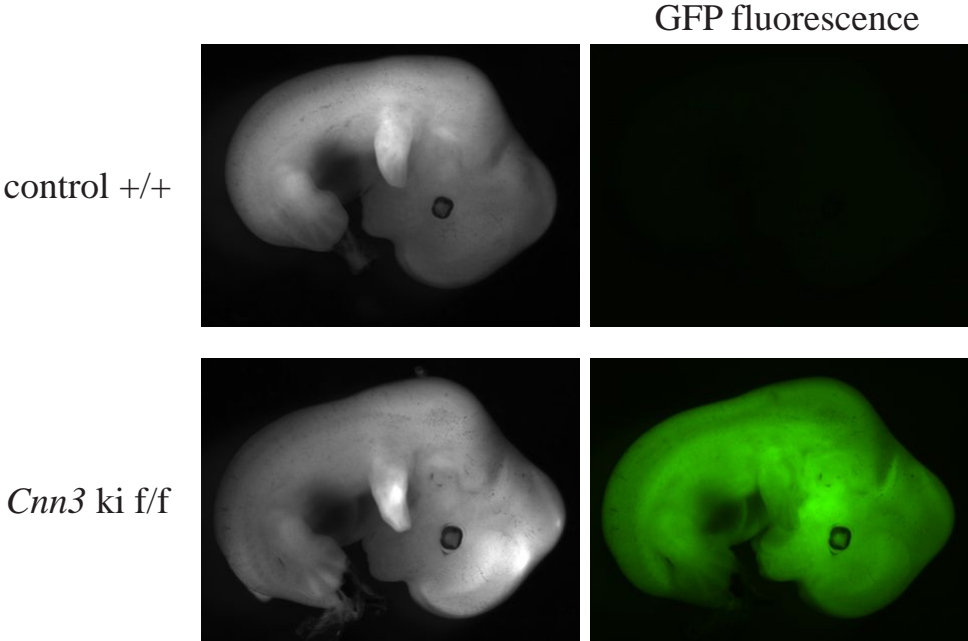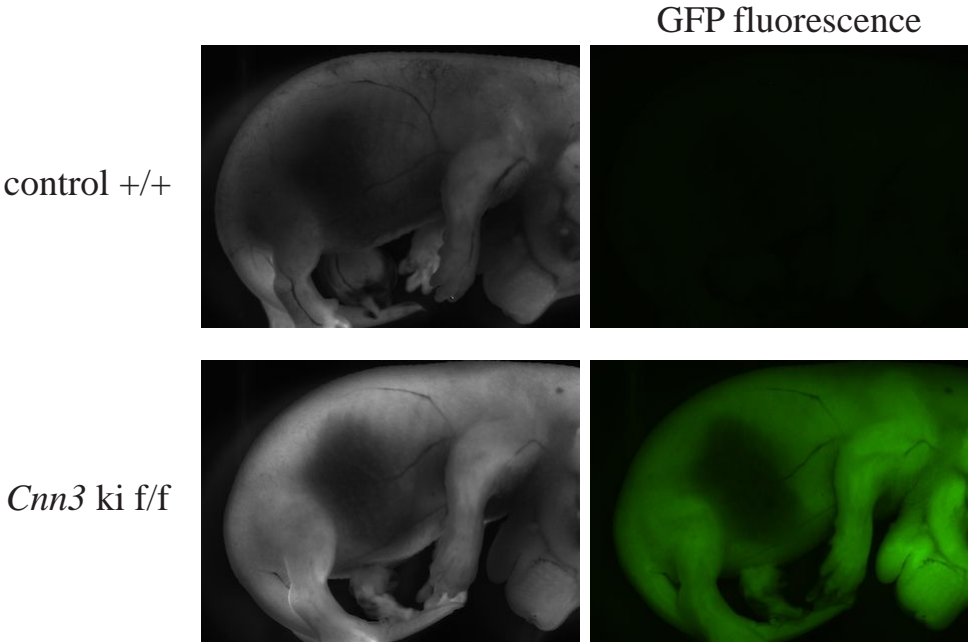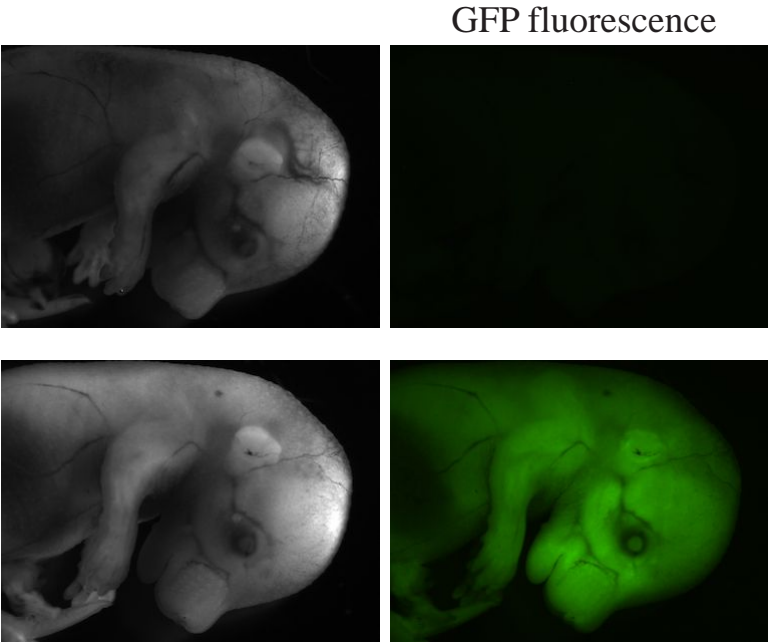

Supplement: S2 Fig — Reflected light images (left) and GFP fluorescence (right panels) in Cnn3 ki f/f and +/+ embryos. (PDF) [file pone.0128385.s002.pdf]

Figure S3

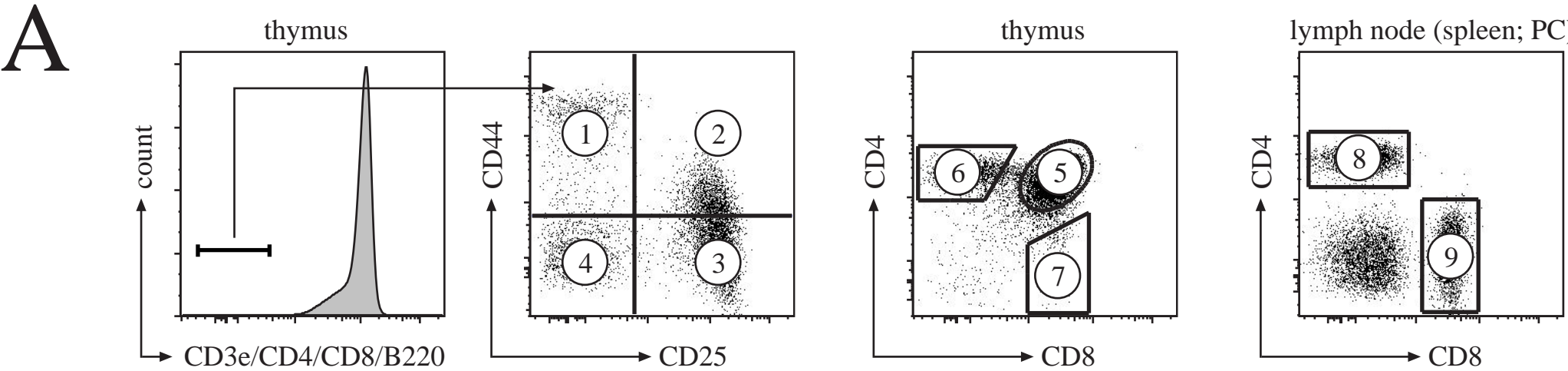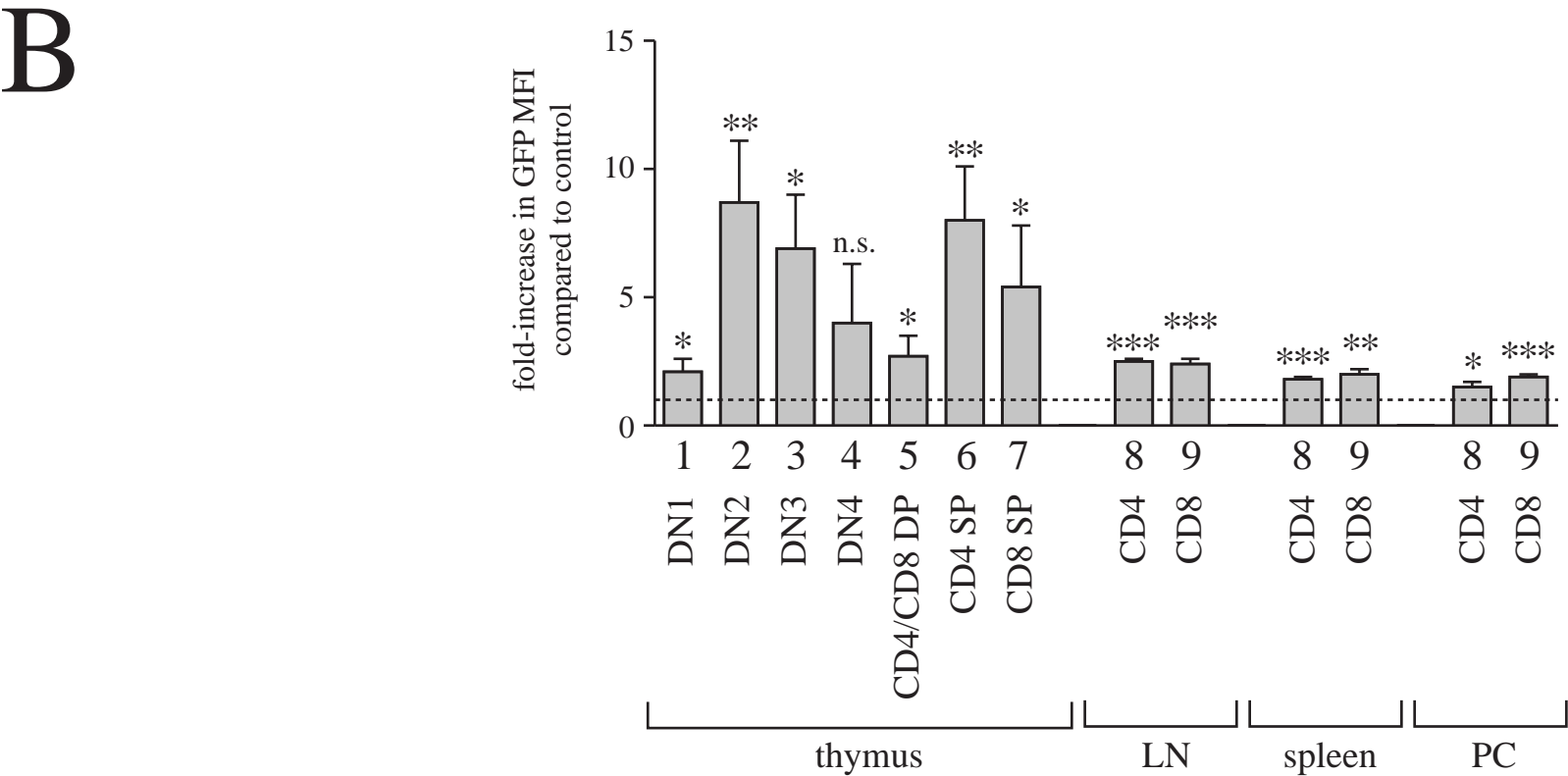

Supplement: S3 Fig — A. Staining pattern and gating strategies for cells isolated from the thymus, lymph nodes, the spleen and the peritoneal cavity. For the identification of early T cell progenitors, thymic cells were stained for CD3e, B220, CD4 and CD8, and lineage-negative cells were further subdivided according to their expression of CD44 and CD25, respectively. Numbers indicate the respective populations as analyzed in B. T cell expression of calponin-3-GFP in different tissues and different developmental stages derived from a Cnn3 ki f/f mouse or a +/+ littermate. In analogy to Fig 4, bar graphs depict the ratio of the GFP comparing ki f/f cells versus +/+ cells. Data represent 4 independent experiments. For statistical analysis, normalized GFP MFI values of control and ki f/f littermates were compared by a paired t-test (p>0.05 = not significant (n.s.), p≤0.05 = *, p≤0.01 = **, p≤0.001 = ***). LN, lymph node; PC, peritoneal cavity; SP, single-positive; DP, double-positive. (PDF) [file pone.0128385.s003.pdf]

Figure S4

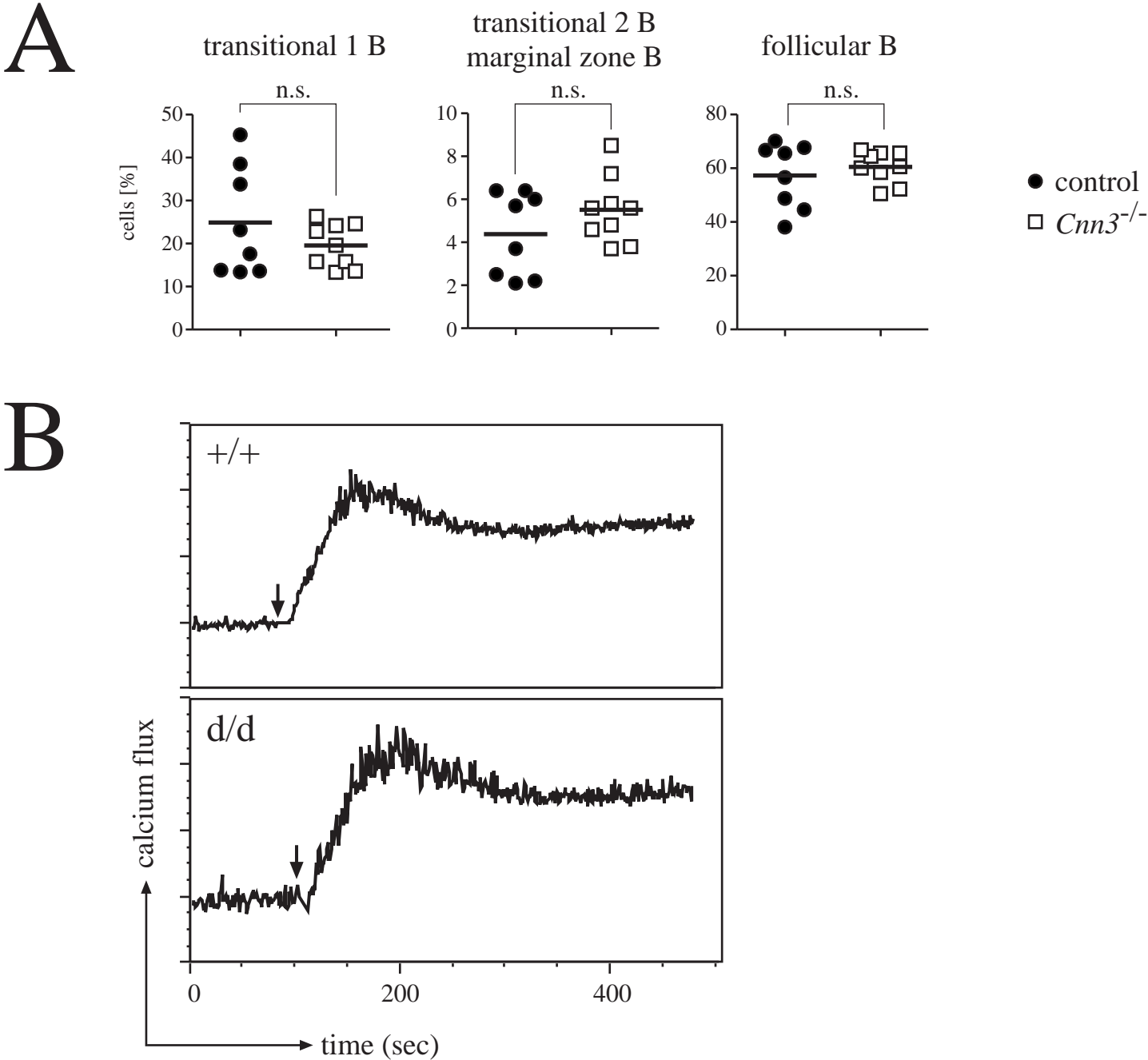

Supplement: S4 Fig — A. Percentages of different developmental stages and cell types derived from the spleen (according to Fig 4A) of control and B cell-specific Cnn3 knockout mice. Controls (+/+ or +/f, positive for mb1-Cre) are depicted as black dots, knockout animals (f/d or f/f by tail PCR, positive for mb1-Cre) as white squares. Individual percentages are calculated on basis of IgM-positive cells. Black bars mark the averaged percentage of cells for each subgroup. Percentages of cells in control and knockout animals were compared in an unpaired t-test (p>0.05 = not significant, n.s.). B. Induced calcium flux in splenic B cells isolated from control and knockout mice. Cells were counterstained with anti-CD43 to exclude T cells, loaded with Indo-1, stimulated with anti-kappa (marked by arrow) and analyzed by flow cytometry. (PDF) [file pone.0128385.s004.pdf]

Figure S5

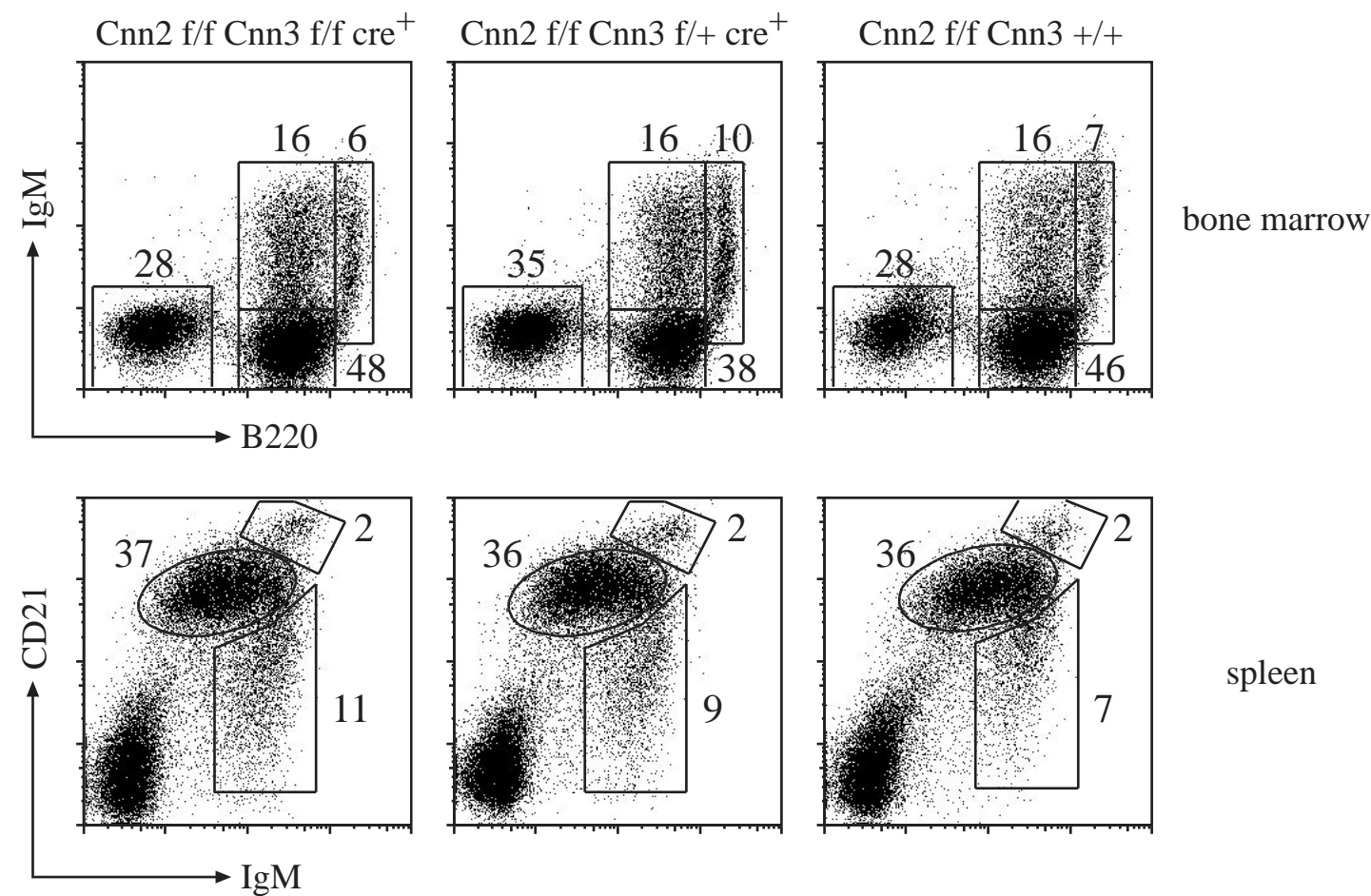

Supplement: S5 Fig — Comparison of bone marrow (upper row) and splenic (lower row) B cell populations in a calponin 2/calponin-3-double deficient mouse (f/f,f/f,mb1-cre+) compared to a calponin 2-deficient (f/f,+/f,mb1-cre+) and a wild type littermate (f/f,+/+). Numbers indicate the percentage of cells in the respective region. (PDF) [file pone.0128385.s005.pdf]
