# Supplementary material for: A Conditional Knockout Mouse Model Reveals That Calponin-3 Is Dispensable for Early B Cell Development
Source: PLoS One. 2015 Jun 5;10(6):e0128385. doi: 10.1371/journal.pone.0128385 (PMC4457629; doi:10.1371/journal.pone.0128385)
Supplement: S1 Supplementary Materials — (PDF) [file pone.0128385.s006.pdf]

sequence of targeting vector

GTGAACCATCACCCTAATCAAGTTTTTTGGGGTCGAGGTGCCGTAAAGCACTAAATC
GGAACCCTAAAGGGAGCCCCCGATTTAGAGCTTGACGGGGAAAGCCGGCGAACGTGG
CGAGAAAGGAAGGGAAGAAAGCGAAAGGAGCGGGCGCTAGGGCGCTGGCAAGTGTA
GCGGTCACGCTGCGCGTAACCACCACACCCGCCGCGCTTAATGCGCCGCTACAGGGCG
CGTCCATTCGCCATTCAGGCTGCGCAACTGTTGGGAAGGGCGATCGGTGCGGGCCTCT
TCGCTATTACGCCAGCTGGCGAAAGGGGGATGTGCTGCAAGGCGATTAAGTTGGGTA
ACGCCAGGGTTTTCCCAGTCACGACGTTGTAAAACGACGGCCAGTGAATTGTAATAC
GACTCACTATAGGGCGAATTGGGCCCTCTAGATGCATGCTCGAGCGGCCGCCAGTGT
GATGGATCTGAAGTAAATTATCCATTATCCATTCCCGTTTCTCTAATTATGGGTAAT
GGAGTGTGAAGGGGACACAGTAATCCCTGAAATCATGTTTACTTCAGCGGAGCCGCC
CCTTGGTGATGGGGGAGGGGAGGCACCTGCCAGCGCCAGCAGCTCTGACACTTTTCCG
GGGCTATCTGAAAGCTTTCATGAGCCTACAAGAGGAGTGTGACTCTTTCGTTTATTT
GACGTGAGAAATGACATCTTTGTTCACCTTGAGGGAAGACATGAAAAGCGGCCATTG
TCGTGTCCTGTCTACTGAAGAGGAACCACAGTCAAGTGTGGTCAGTTGAGCTCATTT
CTTTTTAAGACTCTGCTGACCTTCGTTGTTAGACATAAGCTCGCCAGCTTCTTACCTG
TCCACACTGGCCCTGATGACCCACGACACACATGCAGCCTCAAGCCCTCCTCCGTAGC
TGTGCTTGTGTCCTGCCAGGCTAGTTAGACCTGAACTCCAGGGACCTCCCCCTGAGCA
ATGTAGTGATGTGCCTGGCTCAGTAATTAAGCCTAATTATGTTTTATGGCGAGCAGA
CATCCCCGCTCTTCATTTCCATTTCTGACAGTTTAGTAATTGATGCCCAAGCTCCTAT
CTAGCCCCTGGGCTCAGCCTAAGTGTGTCTGCTTGGCATTTTTAGCTGTGCTGAATGA
AAAGGCAAGAGTACATGATCTCGCCTCGCCTCCAGCTGCCAGCCCTGGCTGTTCTGGA
ACTCACTCTGTAGACCAGACTGGCCTCAGTCTCCCAAGATCCACTTGCCTCTGCCTAC
TGAGCTGGGATTAAAGGTGTGCTGCATGCCGTTTTAAAATATTTGTTCCACTTTTTT
AAAAGTTTAGAGTGGCTGGGCAGTGGTGGCACAGGCCTTTTAATCCCAGCACTCGGG
AGGCAGAGGCAGGCGGATTTTTGAGTTCGAGGCCAGCCTGGTNTTCCGAGGAGTGAG
TTCCAGGGACAGCAATGGATACACAGAGAAACCCTGTCTCAAAAAAAAAAAAAAAAC
AAAAGAAAAAAAAAAAGGGGGGATGGGGGTTAGGTTTATGGTATTGTTGTGAAATTC
AAAGGTAGTTTTTTTAAGTCCTGAGAACTGTTTTTAAAAATGTATGTTTAAAAGTAT
ACGTATTGTGTTTGTGTGCCTGCACACTCGCATACAATGTAATGAATGCAATTGCTG
TAATAAAAGGCGGATCCCAGAACTTTGGTAAACCCCTAGAATGGTATGTTCTAGATA
GTTGAGATACTGTGTTCAATAGTCCCATCTCTCCCTGTCCTTCTTTGGAATGTGGTGA
TTCATGACTCAGTCTTTAAAAGTTTAAGGTCCAGCTGATGGCGTATTGCAACACTCT
GCTAAATCCTGCCCCTTGCGTCATGCGTGACGGGGACTTCGTCTTGTATTGTTGAATT
CTCCCCTTGGTCCCGGCTTCTCTTTTTATGCTCCTTAATGGACACTGCCTTCACTCAC
AGTCTGTGTGTAGTTAAGCTTGGCTGGACGTAAACTCCTCTTCAGACCTAATAACTT
CGTATAGCATACATTATACGAAGTTATATTAAGGGTTATTGAATATGATCGGAATTA
TCGAATTCCCTGCAACAGCTGGCTACAGTCACCAGCAAACTCTCCTGCGTGGTGGGGA
GAGCTACCTGTCAGAGCTGGGGCTTAGTGGACAATGAACGAAAGCAGATGGCATTGT
GAGCCAGTGGTGTGTGTTCCCTGTTACTGAGTCCTTCTGAAATCCCGGAACTTGTATT
TCAGATTGCATCCAAGTATGACCAGCAGGCCGAGGAAGATCTGCGCAACTGGATAGA
AGAGGTGACAGGCCTAGGCATTGGCACCAACTTCCAGTTGGGGCTGAAGGACGGCAT
CATATTGTGCGAACTCATAAACAAGCTACAGCCAGGCTCTGTGAAGAAAGTCAACGA
ATCCTCACTAAATTGGCCCCAGTTGGAGAATATCGGCAACTTCATTAAAGCTATCCA
GGCTTACGGTATGAAGCCCCATGATATATTTGAAGCAAACGACCTCTTTGAGAATGG
CAACATGACCCAGGTTCAGACGACGCTGGTGGCTCTAGCAGGTCTGGCGAAAACAAA
AGGATTCCATACAACCATTGACATTGGCGTTAAGTATGCAGAAAAACAAACAAGACG
TTTTGATGAAGGCAAATTAAAGGCTGGCCAGAGTGTAATTGGTTTACAGATGGGTAC

CAACAAATGTGCCAGCCAGGCGGGCATGACAGCCTATGGGACTCGGAGGCATCTTTA
TGATCCCAAGATGCAGACGGACAAACCCTTTGACCAGACCACGATTAGCCTGCAGAT
GGGCACCAACAAAGGGGCCAGCCAGGCTGGGATGTTAGCACCGGGCACCAGAAGAGA
CATCTATGACCAGAAGCTGACATTACAGCCAGTGGACAACTCGACCATTTCTCTACA
GATGGGCACCAACAAAGTTGCTTCCCAGAAAGGAATGAGCGTGTATGGGCTTGGGCG
GCAAGTATATGACCCCAAGTACTGTGCCGCACCCACAGAACCTGTCATTCACAACGGA
AGCCAGGGCACGGGCACCAATGGGTCGGAAATCAGTGATAGCGATTATCAGGCAGAA
TACCCCGATGAATATCATGGCGAGTACCCAGACGACTACCCTCGGGAGTACCAGTAT
GGCGACGACCAGGGCATTGATTATTTACCGGTCGCCACCATGGTGAGCAAGGGCGAG
GAGCTGTTCACCGGGGTGGTGCCCATCCTGGTCGAGCTGGACGGCGACGTAAACGGCC
ACAAGTTCAGCGTGTCCGGCGAGGGCGAGGGCGATGCCACCTACGGCAAGCTGACCCT
GAAGTTCATCTGCACCACCGGCAAGCTGCCCGTGCCCTGGCCCACCCTCGTGACCACC
CTGACCTACGGCGTGCAGTGCTTCAGCCGCTACCCCGACCACATGAAGCAGCACGACT
TCTTCAAGTCCGCCATGCCCGAAGGCTACGTCCAGGAGCGCACCATCTTCTTCAAGGA
CGACGGCAACTACAAGACCCGCGCCGAGGTGAAGTTCGAGGGCGACACCCTGGTGAA
CCGCATCGAGCTGAAGGGCATCGACTTCAAGGAGGACGGCAACATCCTGGGGCACAA
GCTGGAGTACAACTACAACAGCCACAACGTCTATATCATGGCCGACAAGCAGAAGAA
CGGCATCAAGGTGAACTTCAAGATCCGCCACAACATCGAGGACGGCAGCGTGCAGCT
CGCCGACCACTACCAGCAGAACACCCCCATCGGCGACGGCCCCGTGCTGCTGCCCGAC
AACCACTACCTGAGCACCCAGTCCGCCCTGAGCAAAGACCCCAACGAGAAGCGCGATC
ACATGGTCCTGCTGGAGTTCGTGACCGCCGCCGGGATCACTCTCGGCATGGACGAGCT
GTACAAGTAAGTCGAGCCTCTAGAACTATAGTGAGTCGTATTACGTAGATCCAGACA
TGATAAGATACATTGATGAGTTTGGACAAACCACAACTAGAATGCAGTGAAAAAAAT
GCTTTATTTGTGAAATTTGTGATGCTATTGCTTTATTTGTAACCATTATAAGCTGCA
ATAAACAAGTTAACAACAACAATTGCATTCATTTTATGTTTCAGGTTCAGGGGGAGG
TGTGGGAGGTTTTTTAATTCGCGGCCGCCAGTGTGATGGATGACGGTATCGATAAGC
TTGATCCTTAATTAAGGAGAGGTTTTCACCGTCATCACCGAAACGCGCGAGGCAGCC
TCGAGGGAGCTTCAAAAGCGCTCTGGAAGTTCCTATACTTTCTAGAGAATAGGAACT
TCGGAATAGGAACTTCAAGATCCCCCTGGCGAAAGGGGGATGTGCTGCAAGGCGATT
AAGTTGGGTAACGCCAGGGTTTTCCCAGTCACGACGTTGTAAAACGACGGCCAGTGA
ATTCGAGCTCTCCCATATGGTCGAGCAGTGTGGTTTTGCAAGAGGAAGCAAAAAGCC
TCTCCACCCAGGCCTGGAATGTTTCCACCCAATGTCGAGCAGTGTGGTTTTCAAGAGG
AAGCAAAAAGCCTCTCCACCCAGGCCTGGAATGTTTCCACCCAATGTCGAGCAAACC
CCGCCCAGCGTCTTGTCATTGGCGAATTCGAACACGCAGATGCAGTCGGGGCGGCGCG
GTCCCAGGTCCACTTCGCATATTAAGGTGACGCGTGTGGCCTCGAACACCGAGCGAGC
GACCCTGCAGCCAATATGGGATCGGCCATTGAACAAGATGGATTGCACGCAGGTTCT
CCGGCCGCTTGGGTGGAGAGGCTATTCGGCTATGACTGGGCACAACAGACAATCGGC
TGCTCTGATGCCGCCGTGTTCCGGCTGTCAGCGCAGGGGCGCCCGGTTCTTTTTGTCA
AGACCGACCTGTCCGGTGCCCTGAATGAACTGCAGGACGAGGCAGCGCGGCTATCGT
GGCTGGCCACGACGGGCGTTCCTTGCGCAGCTGTGCTCGACGTTGTCACTGAAGCGGG
AAGGGACTGGCTGCTATTGGGCGAAGTGCCGGGGCAGGATCTCCTGTCATCTCACCT
TGCTCCTGCCGAGAAAGTATCCATCATGGCTGATGCAATGCGGCGGCTGCATACGCTT
GATCCGGCTACCTGCCCATTCGACCACCAAGCGAAACATCGCATCGAGCGAGCACGTA
CTCGGATGGAAGCCGGTCTTGTCGATCAGGATGATCTGGACGAAGAGCATCAGGGGC
TCGCGCCAGCCGAACTGTTCGCCAGGCTCAAGGCGCGCATGCCCGACGGCGAGGATCT
CGTCGTGACCCATGGCGATGCCTGCTTGCCGAATATCATGGTGGAAAATGGCCGCTTT
TCTGGATTCATCGACTGTGGCCGGCTGGGTGTGGCGGACCGCTATCAGGACATAGCG
TTGGCTACCCGTGATATTGCTGAAGAGCTTGGCGGCGAATGGGCTGACCGCTTCCTCG
TGCTTTACGGTATCGCCGCTCCCGATTCGCAGCGCATCGCCTTCTATCGCCTTCTTGA

CGAGTTCTTCTGAGGGGATCGGCAATAAAAAGACAGAATAAAACGCACGGGTGTTGG
GTCGTTTGTTCGGATCCGTCGAGGGAGCTTCAAAAGCGCTCTGAAGTTCCTATACTT
TCTAGAGAATAGGAACTTCGGAATAGGAACTTCAAGATCCCCCTGGCGAAAGGGGGA
TGTGCTGCAAGGCGATTAAGTTGGGTAACGCCAGGGTTTTCCCAGTCACGACGTTGT
AAAACGACGGCCAGTGAATTCGAGCTCTCCCATATGGTCGAGACGGTATCGATAAGC
TTGAGATCCTAAGCTTGGCTGGACGTAAACTCCTCTTCAGACCTAATAACTTCGTAT
AGCATACATTATACGAAGTTATATTAAGGGTTATTGAATATGATCGGAATTATCGAA
TTCCTGCAGCCCATCTGCAGAATTCGCCCTTCTCGGAGGCATCTTTATGATCCCAAGA
TGCAGACGGACAAACCCTTTGACCAGACCACGATTAGCCTGCAGATGGGCACCAACA
AAGGGGCCAGCCAGGTAAGCGGGGTCTTTTTAACACTTGAAGCAAAGGGCGCGGAAG
GGTATGCTAAGTTAGTGCTGACCCAATAGTGAGTAGCTGGACTCTCAAATGGTATTT
CTTCCATGGTATTTTTAGTCTTCAGGAAAGTAGATTTTGCTGAGAGAGCAAAATAAT
CAAAGGGCCCTTTCTGCAGCCTAAAAAGTGTAGCGTGACTAATGGTTTGGTTGGCAG
TAAGATCAGCTGGCCTTTCTGCCTAACCAGAAGAAAAAAGTGATGTTTATATATGTT
CCTGCTCCAAAAGCCAAGGAAAGGATAGTTGGTTCTCCCCTGCCCCATTTTGGTTTTG
TTTTTAAAGGGGCCCATTTAGGTGGATATGTAAGATTATTTGGCTGTTTGGTAAGAG
CTGTTTAATCAGAATTTTTATGAAACATTACGGTAGCAGAAATGAATTCATATGAAA
TGGTAGTTAAATAATTTTTAAAGTAGTGGGGAGGAAAGGGTTTGTTCGGCTTACGTT
TCCATGCCATAGGCCAACCGAGGCAGGAACCTGGAGGCAGGAGCTGAAGCAGAGAGG
AGTGTCACCTGCTGGCTTCCCTTGCTTGCTCTGCTACCGTTCTTAGACAGCCAGGCCT
ACCTGCCTGGGAGCCTCCAGCATCCATTAGCACTTCAGAAAATGCCCCACAGACTCTC
GCACAGGCCAATCTGATAGAGCTAATTATTTCATTGAGGTCTACAGTTCCCAGGTGT
GTCTACCTGAAAGCCAAAATTAGTCACCACAGGTTTTGAGATAGATGAGGGTGTGGA
CACGATGGGGGAGAGAGGGACAGGCCGGGTAAAGGCGGTTATCCGGTGGCTTGAGTT
TGATCCCTGGAACCCATGAAGACGGAAGGAGAGGGCGACTCTACAACATTGTTCTTC
AGCCTTCACATTCACATAACCCTGGACTCTGGACCTAGGTTTGTCTAGTGCCCTCTCC
ACTCCTCCCCACCCCCACACACAAATAGTAAAAACAGTGAAATACCAGGAATTGTTT
GAAAGCATCAGGATTTTATATCTAAAAATCGTGGGACGGAGTTTTGGTAAGCTTTCC
TGTTTTTCCAGTGACATCTGTCGTTCCCCGTGTGGAGCGGAGCGCTCCGGGTCACCTG
CAGTTGGTTCTCTTGGCGGTCTCTCGTGTTTGCCTTTTCACTCACTCACCTCTCAAGT
GTGTGCTTGACAGCAGTGGGTGCTGAGGTGTGGGTTACCCACTGTGAAAGGGAGAGA
GGAGCAGGTCTGAGGTGGGACTGTGGGTGGGGGAGATCTTGTGTGGCAGCCTCTTGA
GAAGACTTGGTCCCTGAGATCTGCTCAGCCTGGTCTTGTTGAAGGCATGGATGCTTT
TTTACAATTGGAGACCTGGGTGGTCATTAATAGACCATGTTCATATGTAATTCAGCA
ATATGTGTAAAAAAATGTAGTTTATCTGATTTTTTTTATGTGTCTAGCTATTTCGCC
TGTGTGTATGTATATGAGCCTTGTATATGCCTGCTACCCTTGGAAGTCAGGAATGGA
TGGATGCCCTGAAACTGAGTTACAGTTTAGGTGCTGGGAACTGGTCCTGGGTCCTCT
GCAAGACAGACAAGTGCTCTTAACCACAGAGCTCTTGTCGCCAGCCCTGGGCTTGGTT
TTCCTTTGTCCCTCACTTAGTAGACTGACGTATTGCTTCTGTATTACATATAGGAAG
TTAAACTCAAGGTAGGTTAATTTGTCTGACACCATATAGTGATCTTAACAGTGGGCA
GAGATTTGAAGGAAGAAGGCCTGGCTCAACTCAGCAGCTCAAGAGCCAACGAATCCC
GTCATAAATATGAGTGGTATAAGAATATTTTAGTAAGTTAGGGTCACGTCAGAACCA
ATTGAAAGTTTTTATGTTAATGCATTCTGTGTGAATTTAATAATTTAGACAGTGTTA
AGAAGTATAGATCTTATCTGTTATCTTACCTTGTGTACCATTATGCTCTAATACCAG
GCTGGGATGTTAGCACCGGGCACCAGAAGAGACATCTATGACCAGAAGCTGACATTA
CAGCCAGTGGACAACTCGACCATTTCTCTACAGATGGGCACCAACAAAGTTGCTTCCC
AGAAAGGAATGAGCGTGTATGGGCTTGGGCGGCAAGTATATGACCCCAAGTACTGTG
CCGCACCCACAGAACCTGTCATTCACAACGGAAGCCAGGGCACGGGCACCAATGGGTC
GGAAATCAGTGATAGCGATTATCAGGCAGAATACCCCGATGAATATCATGGCGAGTA

CCCAGACGACTACCCTCGGGAGTACCAGTATGGCGACGACCAGGGCATTGATTATTA
GAGTCACACACAGGAGCGCAGTATTTAGTCCATTGTTTTATCCAGTGAGACCCAAGC
TAGCCTTGAATAATTCTTCTCTCGTCTTCCTGAAACACTATTATGCTTGTTGTACCTT
TAAAGTATGCCTTATGTACATTCCTTTCTCCTTTTCCTGCCTCCTCCCTAAATAGCTG
CCTTCTAGTGCTGTAGCAAGGGAGCCCTACTGCATAGCCAGTAACTCGCGTCTGTACC
ATGGAAAGGGCGGAACGATTCTCCAGGACAGCCAGCTCTTTCGTTGAAGATCTATCT
ATGCATTTTTTTACACTTACACATAAACTGGTATTTTCGAACAATAGGAAACTATTT
TTTCTCCTTTTTTTACAGTTTTAGTACGTATCTGGCTTGTATGTGGAAGACTAAAAA
GTTGATTTGCTAAATGTGGTCTTTGCCAACTAAAATCTGAGATGCAGCTTTAGACCC
TGACACGTGGATGTTCTTCTGCAGTCTTGTCTGCTAAGTTTTAAATAAAGTCATGAT
CAGTGTGCATTTGTGATTACATGTGTACTCATTCTTTTCCCAAGCTGACGAGGTCTCT
CCCGAGTGGCGCTTCGAAAGGCGTGCATGCAGAAATGGCCGAGGACATGCAGGTTTG
GGTGGTGTGCCTGCAGACTTCATTTGTGCCAATGTATTACTGTAGAGTCGCTCTGTT
TCCTTCAACTGTATTTATTGCTGCGTTTCTCAAAGGGCGAATTCCAGCACACTGGCGG
CCGTTACTAGTGGATCCGAGCTCGGTACCAAGCTTGGCGTAATCATGGTCATAGCTG
TTTCCTGTGTGAAATTGTTATCCGCTCACAATTCCACACAACATACGAGCCGGAAGC
ATAAAGTGTAAAGCCTGGGGTGCCTAATGAGTGAGCTAACTCACATTAATTGCGTTG
CGCTCACTGCCCGCTTTCCAGTCGGGAAACCTGTCGTGCCAGCTGCATTAATGAATCG
GCCAACGCGCGGGGAGAGGCGGTTTGCGTATTGGGCGCTCTTCCGCTTCCTCGCTCAC
TGACTCGCTGCGCTCGGTCGTTCGGCTGCGGCGAGCGGTATCAGCTCACTCAAAGGCG
GTAATACGGTTATCCACAGAATCAGGGGATAACGCAGGAAAGAACATGTGAGCAAAA
GGCCAGCAAAAGGCCAGGAACCGTAAAAAGGCCGCGTTGCTGGCGTTTTTCCATAGG
CTCCGCCCCCCTGACGAGCATCACAAAAATCGACGCTCAAGTCAGAGGTGGCGAAACC
CGACAGGACTATAAAGATACCAGGCGTTTCCCCCTGGAAGCTCCCTCGTGCGCTCTCC
TGTTCCGACCCTGCCGCTTACCGGATACCTGTCCGCCTTTCTCCCTTCGGGAAGCGTG
GCGCTTTCTCATAGCTCACGCTGTAGGTATCTCAGTTCGGTGTAGGTCGTTCGCTCCA
AGCTGGGCTGTGTGCACGAACCCCCCGTTCAGCCCGACCGCTGCGCCTTATCCGGTAA
CTATCGTCTTGAGTCCAACCCGGTAAGACACGACTTATCGCCACTGGCAGCAGCCACT
GGTAACAGGATTAGCAGAGCGAGGTATGTAGGCGGTGCTACAGAGTTCTTGAAGTGG
TGGCCTAACTACGGCTACACTAGAAGAACAGTATTTGGTATCTGCGCTCTGCTGAAG
CCAGTTACCTTCGGAAAAAGAGTTGGTAGCTCTTGATCCGGCAAACAAACCACCGCT
GGTAGCGGTGGTTTTTTTGTTTGCAAGCAGCAGATTACGCGCAGAAAAAAAGGATCT
CAAGAAGATCCTTTGATCTTTTCTACGGGGTCTGACGCTCAGTGGAACGAAAACTCA
CGTTAAGGGATTTTGGTCATGAGATTATCAAAAAGGATCTTCACCTAGATCCTTTTA
AATTAAAAATGAAGTTTTAAATCAATCTAAAGTATATATGAGTAAACTTGGTCTGAC
AGTTACCAATGCTTAATCAGTGAGGCACCTATCTCAGCGATCTGTCTATTTCGTTCAT
CCATAGTTGCCTGACTCCCCGTCGTGTAGATAACTACGATACGGGAGGGCTTACCATC
TGGCCCCAGTGCTGCAATGATACCGCGAGACCCACGCTCACCGGCTCCAGATTTATCA
GCAATAAACCAGCCAGCCGGAAGGGCCGAGCGCAGAAGTGGTCCTGCAACTTTATCC
GCCTCCATCCAGTCTATTAATTGTTGCCGGGAAGCTAGAGTAAGTAGTTCGCCAGTT
AATAGTTTGCGCAACGTTGTTGCCATTGCTACAGGCATCGTGGTGTCACGCTCGTCGT
TTGGTATGGCTTCATTCAGCTCCGGTTCCCAACGATCAAGGCGAGTTACATGATCCCC
CATGTTGTGCAAAAAAGCGGTTAGCTCCTTCGGTCCTCCGATCGTTGTCAGAAGTAA
GTTGGCCGCAGTGTTATCACTCATGGTTATGGCAGCACTGCATAATTCTCTTACTGTC
ATGCCATCCGTAAGATGCTTTTCTGTGACTGGTGAGTACTCAACCAAGTCATTCTGA
GAATAGTGTATGCGGCGACCGAGTTGCTCTTGCCCGGCGTCAATACGGGATAATACC
GCGCCACATAGCAGAACTTTAAAAGTGCTCATCATTGGAAAACGTTCTTCGGGGCGA
AAACTCTCAAGGATCTTACCGCTGTTGAGATCCAGTTCGATGTAACCCACTCGTGCAC
CCAACTGATCTTCAGCATCTTTTACTTTCACCAGCGTTTCTGGGTGAGCAAAAACAG

GAAGGCAAAATGCCGCAAAAAAGGGAATAAGGGCGACACGGAAATGTTGAATACTCA
TACTCTTCCTTTTTCAATTCAGAAGAACTCGTCAAGAAGGCGATAGAAGGCGATGCG
CTGCGAATCGGGAGCGGCGATACCGTAAAGCACGAGGAAGCGGTCAGCCCATTCGCC
GCCAAGCTCTTCAGCAATATCACGGGTAGCCAACGCTATGTCCTGATAGCGGTCCGCC
ACACCCAGCCGGCCACAGTCGATGAATCCAGAAAAGCGGCCATTTTCCACCATGATAT
TCGGCAAGCAGGCATCGCCATGGGTCACGACGAGATCCTCGCCGTCGGGCATGCGCGC
CTTGAGCCTGGCGAACAGTTCGGCTGGCGCGAGCCCCTGATGCTCTTCGTCCAGATCA
TCCTGATCGACAAGACCGGCTTCCATCCGAGTACGTGCTCGCTCGATGCGATGTTTCG
CTTGGTGGTCGAATGGGCAGGTAGCCGGATCAAGCGTATGCAGCCGCCGCATTGCAT
CAGCCATGATGGATACTTTCTCGGCAGGAGCAAGGTGGGATGACAGGAGATCCTGCC
CCGGCACTTCGCCCAATAGCAGCCAGTCCCTTCCCGCTTCAGTGACAACGTCGAGCAC
AGCTGCGCAAGGAACGCCCGTCGTGGCCAGCCACGATAGCCGCGCTGCCTCGTCCTGC
AGTTCATTCAGGGCACCGGACAGGTCGGTCTTGACAAAAAGAACCGGGCGCCCCTGC
GCTGACAGCCGGAACACGGCGGCATCAGAGCAGCCGATTGTCTGTTGTGCCCAGTCAT
AGCCGAATAGCCTCTCCACCCAAGCGGCCGGAGAACCTGCGTGCAATCCATCTTGTTC
AATCATGCGAAACGATCCTCATCCTGTCTCTTGATCAGATCTTGATCCCCTGCGCCAT
CAGATCCTTGGCGGCAAGAAAGCCATCCAGTTTACTTTGCAGGGCTTCCCAACCTTAC
CAGAGGGCGCCCCAGCTGGCAATTCCGGTTCGCTTGCTGTCCATAAAACCGCCCAGTC
TAGCTATCGCCATGTAAGCCCACTGCAAGCTACCTGCTTTCTCTTTGCGCTTGCGTTT
TCCCTTGTCCAGATAGCCCAGTAGCTGACATTCATCCGGGGTCAGCACCGTTTCTGCG
GACTGGCTTTCTACGTGTTCCGCTTCCTTTAGCAGCCCTTGCGCCCTGAATTTTGTTA
AAATTCGCGTTAAATTTTTGTTAAATCAGCTCATTTTTTAACCAATAGGCCGAAATC
GGCAAAATCCCTTATAAATCAAAAGAATAGACCGAGATAGGGTTGAGTGTTGTTCCA
GTTTGGAACAAGAGTCCACTATTAAAGAACGTGGACTCCAACGTCAAAGGGCGAAAA
ACCGTCTATCAGGGCGATGGCCCACTAC

short homology arm: 465-1965
loxP: 2000-2033
Cnn3 (ex2-7)-GFP: 2241-3908
SV40 poly(A): 3946-4080
FRT: 4266-4313
neoR: 4716-5519
HSV TK poly(A): 5526-5574
FRT: 5606-5653
loxP: 5837-5870
long homology arm: 5937-9086

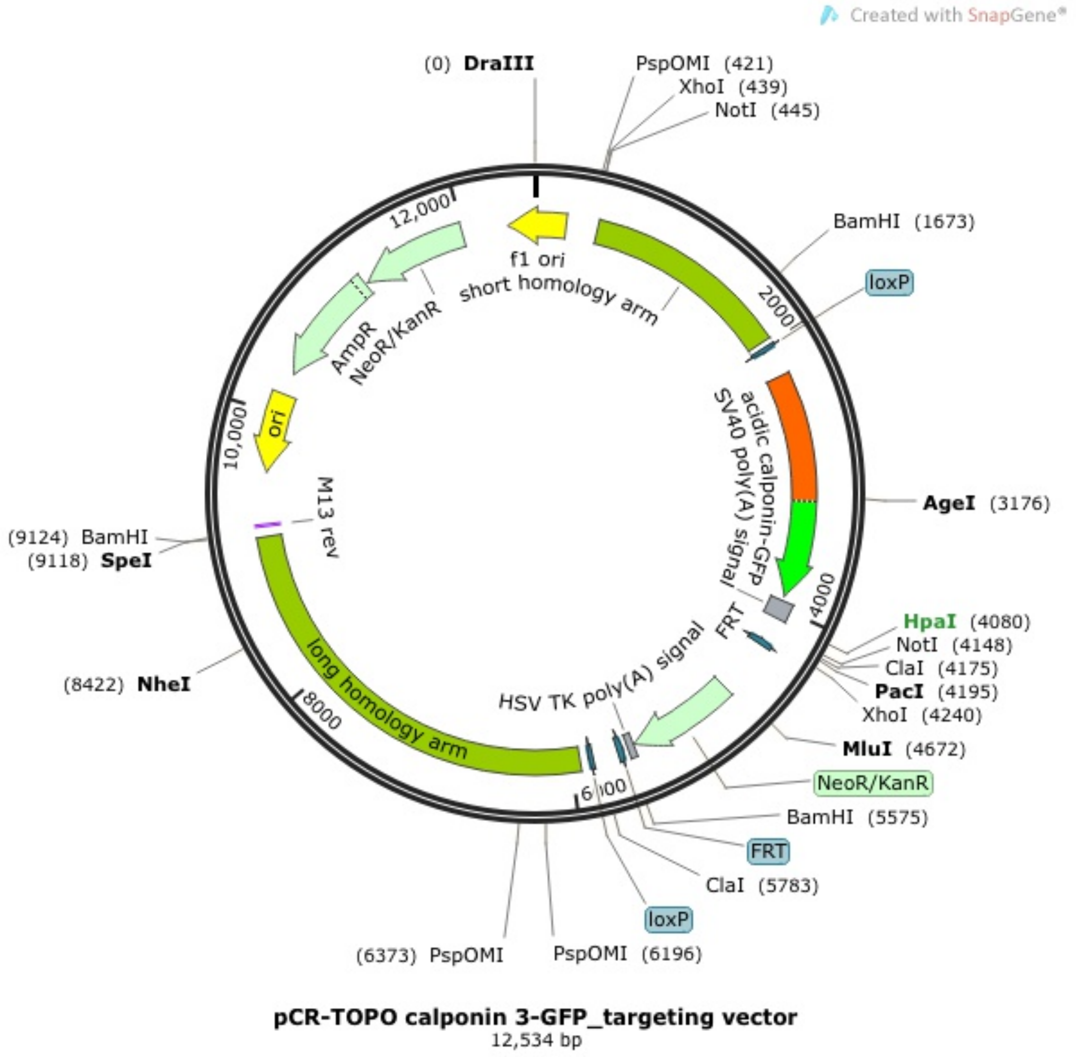

sequence 5' internal probe

GGGATGGGGGTTAGGTTTATGGTATTGTTGTGAAATTCAAAGGTAGTTTTTTTAAGT
CCTGAGAACTGTTTTTAAAAATGTATGTTTAAAAGTATACGTATTGTGTTTGTGTGC
CTGCACACTCGCATACAATGTAATGAATGCAATTGCTGTAATAAAAGGCGGATCCCA
GAACTTTGGTAAACCCCTAGAATGGTATGTTCTAGATAGTTGAGATACTGTGTTCAA
TAGTCCCATCTCTCCCTGTCCTTCTTTGGAATGTGGTGATTCATGACTCAGTCTTTAA
AAGTTTAAGGTCCAGCTGATGGCGTATTGCAACACTCTGCTAAATCCTGCCCCTTGCG
TCATGCGTGACGGGGACTTCGTCTTGTATTGTTGAATTCTCCCCTTGGTCCCGGCTTC
TCTTTTTATGCTCCTTAATGGACACTGCCTTCACTCACAGTCTGTGTGTAGTTAAGCT
TGGCTGGACGTAAACTCCTCTTCAGACCTAATAACTTCGTATAGCATACATTATACG
AAGTTATATTAAGGGTTATTGAATATGATCGGA

sequence 3' external probe

ACCAAGATTTACCTGGTCCTTGTTCTACTACAACTTGGCTGCCCTGGGTTTATCTGAA
ATTCGATTTGGAATTCTCTGAAACGCAAGGGGGAGTTATTACATGTGTCATGTAAAT

GATCCTAAAGCCAGGCATGAGGTGTGCTGTTTGATTAGAGCAGGCTAACCCACAGCT
AAGTGTGGGAAAGGGCAGTGCTGTCACGGGGGAAGCTGCTCGGTCACACCCTCTGAC
CTTGTCTCAGGTCAAGGCTGACTGATGGCCTCGGGTGGTTTTGTAAAGACAGATAAG
AGATGTTGAACCCACAGAAAGGATGTATGCTTGTTCCTCCTGTGGTGGCTTGCTTAG
ATGCTTCTGGGAGTGCCCTGTAAATCACTGTAGGAGACTGGGAGCTAAATGAATTCT
GTGGTACTATTAAAGAAAAAATATCTAAAGTGGTCCTGAGCTGTCAGGCTGTTTCTT
G

genotyping protocol by PCR

primer 1: GGACACTGCCTTCACTCACA
primer 2: CCGTCCTTCAGCCCCAA
primer 3: TTGTTGGTGCCCATCTGCA

Primer 1 and 2 discriminate between the floxed (PCR product of 417 bp) and the wildtype allele (310 bp). Primers 1 and 3 discriminate between the floxed (888 kb) and the deleted allele (254 bp).
